# Fuyuan decoction prevents nasopharyngeal carcinoma metastasis by inhibiting circulating tumor cells/ endothelial cells interplay and enhancing anti-cancer immune response

**DOI:** 10.3389/fphar.2024.1355650

**Published:** 2024-04-25

**Authors:** Yuying Ye, Mengting Lin, Guiyu Zhou, Weiyu Wang, Yinyin Yao, Yafei Su, Jianqiang Qi, Yanfang Zheng, Chunlian Zhong, Xi Chen, Mingqing Huang, Yusheng Lu

**Affiliations:** ^1^ Department of Otorhinolaryngology, Affiliated People’s Hospital (Fujian Provincial People’s Hospital), Fujian University of Traditional Chinese Medicine, Fuzhou, China; ^2^ College of Pharmacy, Fujian Key Laboratory of Chinese Materia Medica, Fujian University of Traditional Chinese Medicine, Fuzhou, China; ^3^ Fujian-Taiwan-Hongkong-Macao Science and Technology Cooperation Base of Intelligent Pharmaceutics, College of Material and Chemical Engineering, Minjiang University, Fuzhou, China; ^4^ Fuzhou Institute of Oceanography, Minjiang University, Fuzhou, China; ^5^ Department of Otorhinolaryngology, Fuzhou Second Hospital, Fuzhou, China; ^6^ Center for Teaching of Clinical Skills, Fujian University of Traditional Chinese Medicine, Fuzhou, China

**Keywords:** traditional Chinese medicine, Fuyuan decoction, cancer metastasis prevention, cell adhesion, transendothelial migration, anti-cancer immune response

## Abstract

Distant metastasis is a major cause of treatment failure in cancer patients and a key challenge to improving cancer care today. We hypothesized that enhancing anti-cancer immune response and inhibiting circulating tumor cells (CTCs) adhesion and transendothelial migration through synergistic multi-target approaches may effectively prevent cancer metastasis. “Fuyuan Decoction” (FYD) is a traditional Chinese medicine compound that is widely used to prevent postoperative metastasis in cancer patients, but its underlying mechanism remains unclear. In this work, we systematically elucidated the underlying molecular mechanism by which FYD prevents cancer metastasis through multi-compound and multi-target synergies *in vitro* and *in vivo*. FYD significantly prevented cancer metastasis at non-cytotoxic concentrations by suppressing the adhesion of CTCs to endothelial cells and their subsequent transendothelial migration, as well as enhancing anti-cancer immune response. Mechanistically, FYD interrupts adhesion of CTCs to vascular endothelium by inhibiting TNF-α-induced CAMs expression via regulation of the NF-κB signaling pathway in endothelial cells. FYD inhibits invasion and migration of CTCs by suppressing EMT, PI3K/AKT and FAK signaling pathways. Moreover, FYD enhances the anti-cancer immune response by significantly increasing the population of Tc and NK cells in the peripheral immune system. In addition, the chemical composition of FYD was determined by UPLC-HRMS, and the results indicated that multiple compounds in FYD prevents cancer metastasis through multi-target synergistic treatment. This study provides a modern medical basis for the application of FYD in the prevention of cancer metastasis, and suggesting that multi-drug and multi-target synergistic therapy may be one of the most effective ways to prevent cancer metastasis.

## 1 Introduction

Cancer is one of the deadliest diseases, and it is estimated that 30.2 million new cancer diagnoses and 16.3 million cancer-related deaths in the word in 2040 (Sung et al., 2021). Metastasis is a critical element leading to the failure of clinical cancer treatment, and more than 90% of cancer-related deaths are related to metastasis (Ferlay et al., 2019). The nasopharyngeal carcinoma (NPC) is a malignant tumor originating from the epithelial cells of the nasopharynx and represents the most prevalent head and neck cancer in South China and Southeast Asia (Chua et al., 2016; Zhang et al., 2022). The incidence rate of NPC has exhibited a significant increase in recent years, with men experiencing notably higher rates of both incidence and mortality compared to women. (Yu et al., 2022). The primary therapeutic approach for patients with advanced NPC involves the utilization of radiotherapy or chemotherapy in combination with radiotherapy; however, the current survival rates remain unsatisfactory due to the emergence of acquired drug resistance and metastasis (Zhong et al., 2018; Zhang et al., 2021). Therefore, it is imperative to explore novel therapeutic and chemopreventive strategies in order to enhance the prognosis of patients afflicted with NPC.

Cancer metastasis is a multi-stage process, mainly including cancer cells shedding from the primary site, entering the vascular microenvironment and migrating to distant tissues and organs (Paul et al., 2017). Cancer cells that enter the blood circulation system are called circulating tumor cells (CTCs). However, the survival of circulating tumor cells (CTCs) in the bloodstream is limited to a small fraction due to mechanical shear stress and immune cell-mediated clearance (Leblanc and Peyruchaud, 2016). Studies have shown that surviving CTCs can establish new tumor lesions and form metastases by adhering to the vascular endothelium and through vascular infiltration (Gay and Felding-Habermann, 2011). Therefore, the adhesion of CTCs to endothelium in the bloodstream microenvironment is a critical step in facilitating distant metastasis (Stott et al., 2010). Cell adhesion molecules (CAMs, including VCAM-1, ICAM-1 and E-selectin), that are involved in the adhesion of CTCs to endothelial cells and are regulated by the NF-κB signaling pathway induced by inflammatory cytokines (Lu et al., 2014; Lu et al., 2019; Liu et al., 2021). The transendothelial migration of CTCs is another critical process facilitating successful metastasis, which is tightly regulated by the epithelial-to-mesenchymal transition (EMT), PI3K/AKT, and FAK signaling pathways (Wang et al., 2022). Furthermore, previous studies have shown that tumor infiltrating cells (TIMs, TILs), along with immunosuppressive checkpoints, play a significant role in immune evasion of NPC cells, ultimately leading to increased growth and metastasis (Al-Rajhi et al., 2020; Liu et al., 2022; Yang et al., 2022). Therefore, based on a comprehensive understanding of the intricate processes underlying cancer metastasis, we proposed that enhancing anti-cancer immune response and inhibiting CTCs adhesion and transendothelial migration through multi-target synergies may effectively prevent cancer metastasis.

Traditional Chinese medicine (TCM) is widely accepted as an effective strategy for the treatment of cancers due to its high efficiency, low toxicity and multiple targets (Yuan et al., 2017; Xiang et al., 2019). In recent years, many herbal monomers have been found to be beneficial in suppressing cancer invasion and metastasis (Wang et al., 2022; Zhong et al., 2023). However, Chinese herbal compounds possessing multi-components and multi-target characteristics may present a more suitable approach for chemoprevention of cancer metastasis, as they aim to enhance the overall therapeutic efficacy in individuals with cancer (Jiang and Liu, 2011; Sarwar et al., 2018; Wang et al., 2020; Yang et al. 2021; Xiangqi Ye and Ye 2021). Fuyuan Decoction (FYD), a traditional Chinese formula comprised of nine herbs (the specific components are presented in the Methods section), has been utilized for years to prevent metastasis of cancer patients after surgery, and has shown favorable clinical response (Xiangqi Ye and Ye 2021). The herbs that make up FYD exhibits many anti-cancer activities. The triterpenoids of *Ganoderma lucidum* can inhibit telomerase activity and prevent the development of NPC (Zheng and Chen, 2017). We recently found that *G. lucidum* extract promotes tumor cell pyroptosis and inhibits metastasis in breast cancer (Zhong et al., 2023). *Ginsenoside Rg3*, a component of Chinese ginseng, can decrease NPC migration and invasion (Wang et al., 2019), and can cause apoptosis in NPC by activating apoptosis-inducing factors (Xie et al., 2013; Law et al., 2014). *Astragalus* polysaccharide triggered apoptosis in nasopharyngeal cancer cells and altered the Bax/bcl-2 ratio and cell cycle (Zhou et al., 2017; Yang et al., 2021). A network correlation analysis revealed that the *Hedyotis* diffusa and *Scutellaria barbata* might improve the survival rate of patients with advanced nasopharyngeal cancer (Han et al., 2020; Wang et al., 2020). Despite numerous studies demonstrating the anti-tumor properties of a single herb in FYD, the precise mechanism underlying the inhibitory effects of FYD compound on cancer metastasis remains elusive.

In this study, we investigated the molecular mechanisms by which FYD prevents NPC metastasis *in vitro* and *in vivo*. *In vitro* experiments, we characterized the chemical composition of FYD and examined its impact on CNE1 cell viability, adhesion, invasion ability, as well as elucidated its underlying molecular mechanism. Additionally, we conducted *in vivo* studies to evaluate the immunomodulatory properties of FYD and its efficacy in preventing cancer metastasis using syngeneic mouse models.

## 2 Materials and methods

### 2.1 Preparation of FYD

The FYD consists of nine different herbs: *Actindia arguta* (Siebold & Zucc.) Planch. ex Miq. [Actinidiaceae; Actindia arguta root] 25 g, *S. barbata* D.Don [Lamiaceae; Scutellaria barbata whole plant] 20 g, *Scleromitrion diffusum* (Willd.) R.J.Wang [Rubiaceae; Scleromitrion diffusum whole plant] 40 g, *Iphigenia indica* (L.) A.Gray ex Kunth [Colchicaceae; Iphigenia indica pseudobulb] 4 g, *Salvia miltiorrhiza* Bunge [Lamiaceae; Salviae miltiorrhizae radix et rhizome] 20 g, *G. lucidum* (Curtis) P. Karst. 15 g, *Panax ginseng* C.A.Mey. [Araliaceae; Panax ginseng radix et rhizome] 12 g, *Lycium chinense* Mill. [Solanaceae; Lycium chinense fruit] 12 g, and *Astragalus mongholicus* Bunge [Fabaceae; Astragalus mongholicus root] 20 g. All herbs were purchased from Jiangxi Kangqingtang Herbal decoction Pieces Co., Ltd. (Zhangshu, China). FYD standard decoction was prepared according to “Management standard of traditional Chinese medicine (TCM) decoction in medical institutions” (Wang et al., 2019). The water-mixed raw materials were soak for 30 min, then decocted for another 45 min and filtered for collection of the filtrate. The residue was decocted again and filtered twice. The filtrates were then freeze-dried to obtain FYD powder.

### 2.2 Composition identification, activity and mechanism analysis of FYD

Compound identification of FYD, cell culturing, cell viability assay, cell cycle assay, cell apoptosis assay, hetero-adhesion assay, migration assay, invasion assay, flow cytometry, western blot, RNA extraction and qRT-PCR assay, *in vivo* pulmonary metastasis assays are described in the online Supplementary Material. The primer sequences for qPCR were presented in Supplementary Table S1.

### 2.3 Animals and ethics statement

Specific pathogen-free female BALB/c mice (20–22 g, 6–8 weeks old) were procured from the Shanghai SLAC Laboratory Animal Co., Ltd (Lu et al., 2019). The mice were housed in a controlled environment with no exposure to pathogens and provided *ad libitum* access to pelleted food and water. All animal experiments adhered to the guidelines outlined in accordance with the Guide for the Care and Use of Laboratory Animals (National Research Council, 1996). All animal experimental procedures were thoroughly evaluated and approved by the Laboratory Animal Ethics Committee of Minjiang University.

### 2.4 Statistical analysis

The results were expressed as means and standard deviations. Statistical analyses were conducted using the SPSS statistical software package. Differences in experimental data were assessed through analysis of variance (ANOVA), with Dunnett’s method employed for adjusting the ANOVA for multiple comparisons. A significance level of *p* < 0.05 was considered statistically significant, while a significance level of *p* < 0.01 was considered extremely statistically significant.

## 3 Results

### 3.1 Compound identification of FYD by UPLC-HRMS technology

**FIGURE 1:**
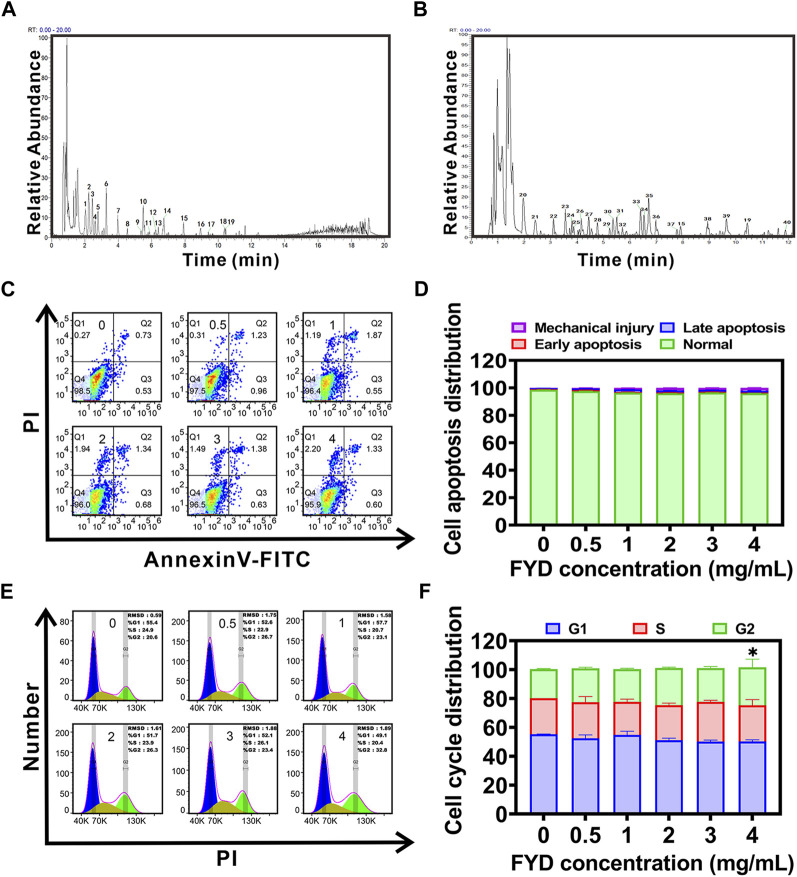
Compound identification and cytotoxicity assessment of Fuyuan Decoction (FYD). **(A, B)** UHPLC-HRMS analysis was performed in positive **(A)** and negative **(B)** ion modes, generating base peak chromatograms (BPC plots) of FYD with the top 40 compounds annotated. **(C, D)** Cell apoptosis was evaluated using flow cytometry after incubating CNE1 cells with FYD for 24 h **(C)**, and the results were presented as a histogram depicting the distribution of apoptotic cells **(D)**. **(E, F)** Cell cycle distribution analysis was carried out using flow cytometry after treating CNE1 cells with FYD for 24 h **(E)**, and the findings were illustrated as a histogram **(F)**. The data are presented as mean ± SD (*n* = 3–6); * indicates *p* < 0.05 compared to the Control.

UPLC-HRMS analysis revealed that FYD comprises a total of 1430 chemical components, which can be classified and identified into 107 categories (Supplementary Table S2). The categories of the top 6 compounds and their subclasses were shown in Supplementary Table S3, and the proportion of the number of the top 6 compounds and their subclasses was shown in Supplementary Figure S1. The peaks exhibiting higher abundance in the positive and negative ion base peak chromatograms (BPC) underwent confirmation of their peak shape and secondary spectral examination prior to being assigned with numerical labels. Sequential numbering was applied to both the positive and negative ion chromatograms (Figures 1A,B). The 40 peaks identified in the positive and negative ion BPC diagram for FYD composition analysis mainly comprise carboxylic acids, purine nucleosides, coumarins, flavonoids, organic oxides, hydroxy acids, cinnamic acids, lipids and other classes of compounds. The detailed information of the compounds was shown in Supplementary Table S4.

### 3.2 Cytotoxicity of FYD

The cytotoxicity of FYD was evaluated in CNE1 cells, revealing a concentration-dependent reduction in cell viability with IC50 and IC10 (Lu et al., 2019) values of 4.695 mg/mL and 4 mg/mL, respectively (Supplementary Figure S2). We also examined the effects of FYD on the proliferative activity of normal cell lines human nasal epithelial cells HNEPC and human umbilical vein endothelial cells (HUVECs), with IC50 values of 5.218 mg/mL and 4.789 mg/mL, respectively (Supplementary Figures S3, S4). Furthermore, based on the results of cell cycle and apoptosis assays, it was observed that medication concentrations equal to or below 4 mg/mL did not exhibit any discernible effect on CNE1 cells, and apoptosis was not produced (Figures 1C–F). These findings suggest that FYD possesses weak cytotoxicity at concentrations less than 4 mg/mL. Therefore, in order to elucidate the mechanism underlying the cancer metastasis preventive effect of FYD, subsequent experiments were conducted using FYD concentrations at or below 4 mg/mL, aiming to demonstrate its inhibitory effect on CTCs adhesion and immune enhancement rather than cytotoxicity-mediated CTCs elimination.

### 3.3 FYD attenuates the adhesion of CNE1 cells to HUVECs by suppressing the expression of inflammatory factor-induced CAMs on endothelial cells

**FIGURE 2:**
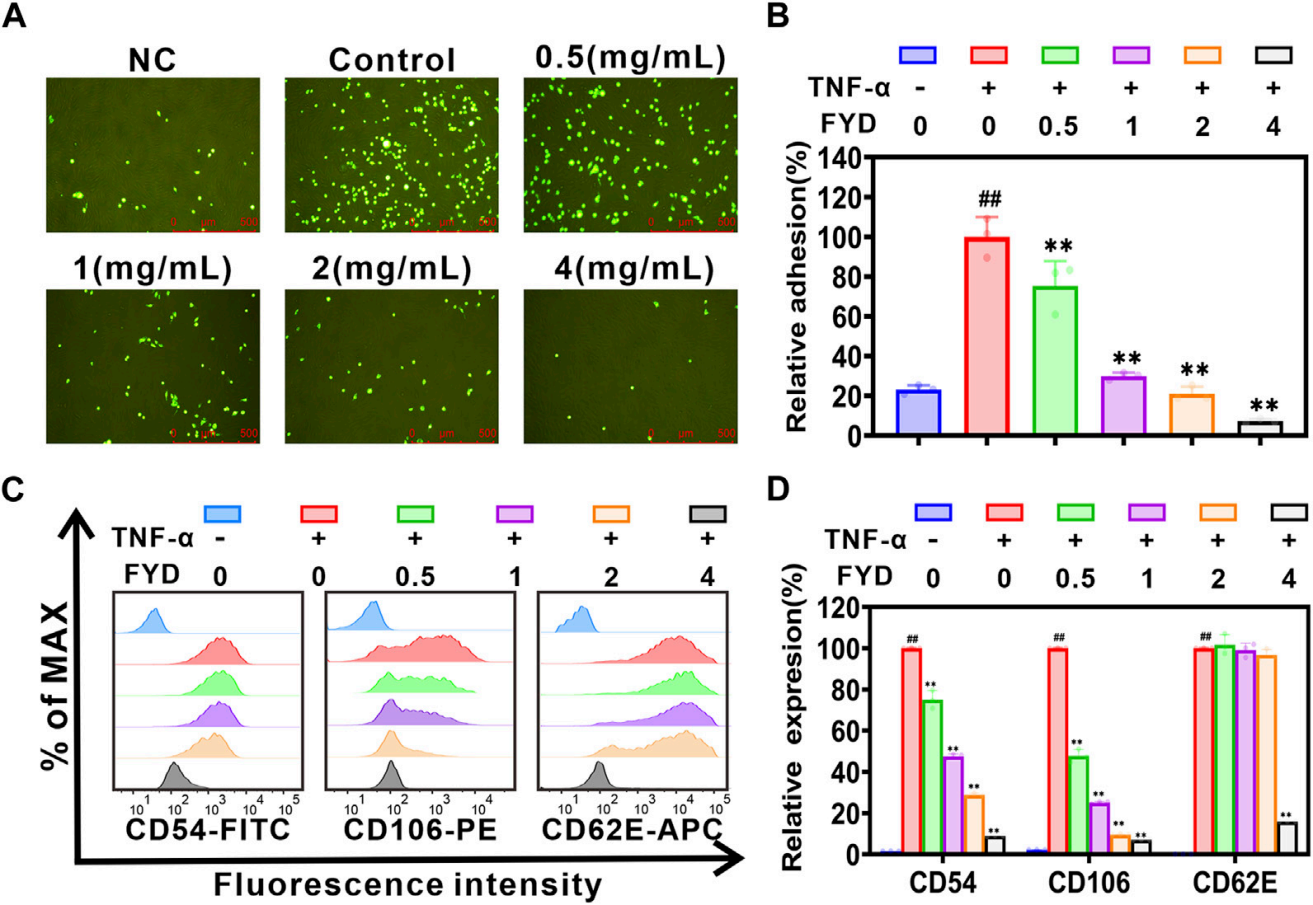
FYD interrupts the adhesion of CNE1 cells to HUVECs by suppressing TNF-α-induced expression of CAMs. **(A)** Fluorescence microscopy was used to capture images of CNE1 cells (labeled with Rhodamine 123, shown in green) adhering to HUVECs. **(B)** FYD significantly attenuated the adhesion of CNE1 cells to HUVECs stimulated by TNF-α. The relative adhesion percentage was calculated based on control conditions (TNF-α only). **(C)** HUVECs were pretreated with FYD for 24 h, followed by incubation with TNF-α (10 ng/mL) for an additional 4 h. The expression of CD106 (VCAM-1), CD54 (ICAM-1), and CD62E (E-selectin) on HUVECs was analyzed using flow cytometry. **(D)** FYD significantly suppressed the expression of VCAM-1, ICAM-1, and E-selectin in TNF-α-stimulated HUVECs. The relative expression of CD106, CD54, and CD62E was quantified as a percentage of the mean fluorescence intensity observed in the control group treated with TNF-α alone. NC, representing the negative control group. The data are presented as mean ± SD (*n* = 3–6); ** indicates *p* < 0.01 compared to the Control (treated with TNF-α alone); ^##^ indicate *p* < 0.01 compared to the Negative control (without TNF-α).

The adhesion of CTCs to endothelial cells is a critical step in the metastasis of cancer cells to distant sites (Stott et al., 2010). Here, we investigated the impact of FYD on the heterogeneous adherence between TNF-α-induced cancer cell CNE1 and HUVECs. The results of the adhesion assay between CNE1 cells and HUVECs showed that FYD effectively inhibited adhesion with a concentration-dependent trend. In the presence of FYD doses of 0.5, 1.0, 2.0, and 4.0 mg/mL, the adhesion rates between CNE1 and HUVECs were observed to be 77.1% ± 12.8%, 30.5% ± 1.8%, 21.5% ± 3.7%, and 7.4% ± 1.1%, respectively, as compared to the control group (induced by TNF-α) (Figures 2A,B). The present study demonstrated that FYD effectively suppressed TNF-α-induced adhesion of CNE1 to HUVECs at non-toxic concentrations.

The pro-inflammatory cytokine TNF-α plays a pivotal role in cancer metastasis by inducing the expression of CAMs on HUVECs (Lian et al., 2016). We employed flow cytometry to assess the effect of FYD on the expression of E-selectin (CD62E), ICAM-1 (CD54), and VCAM-1 (CD106) in HUVECs. The experimental results showed that FYD effectively inhibited the expression of VCAM-1, ICAM-1, and E-selectin in HUVECs with a concentration-dependent trend (Figures 2C,D). This finding prompted us to further investigate the molecular mechanism underlying FYD mediated disruption of CNE1 cells adhesion to endothelial cells in subsequent investigations.

### 3.4 FYD modulates the NF-κB signaling pathway to attenuate the expression of CAMs in HUVECs

**FIGURE 3:**
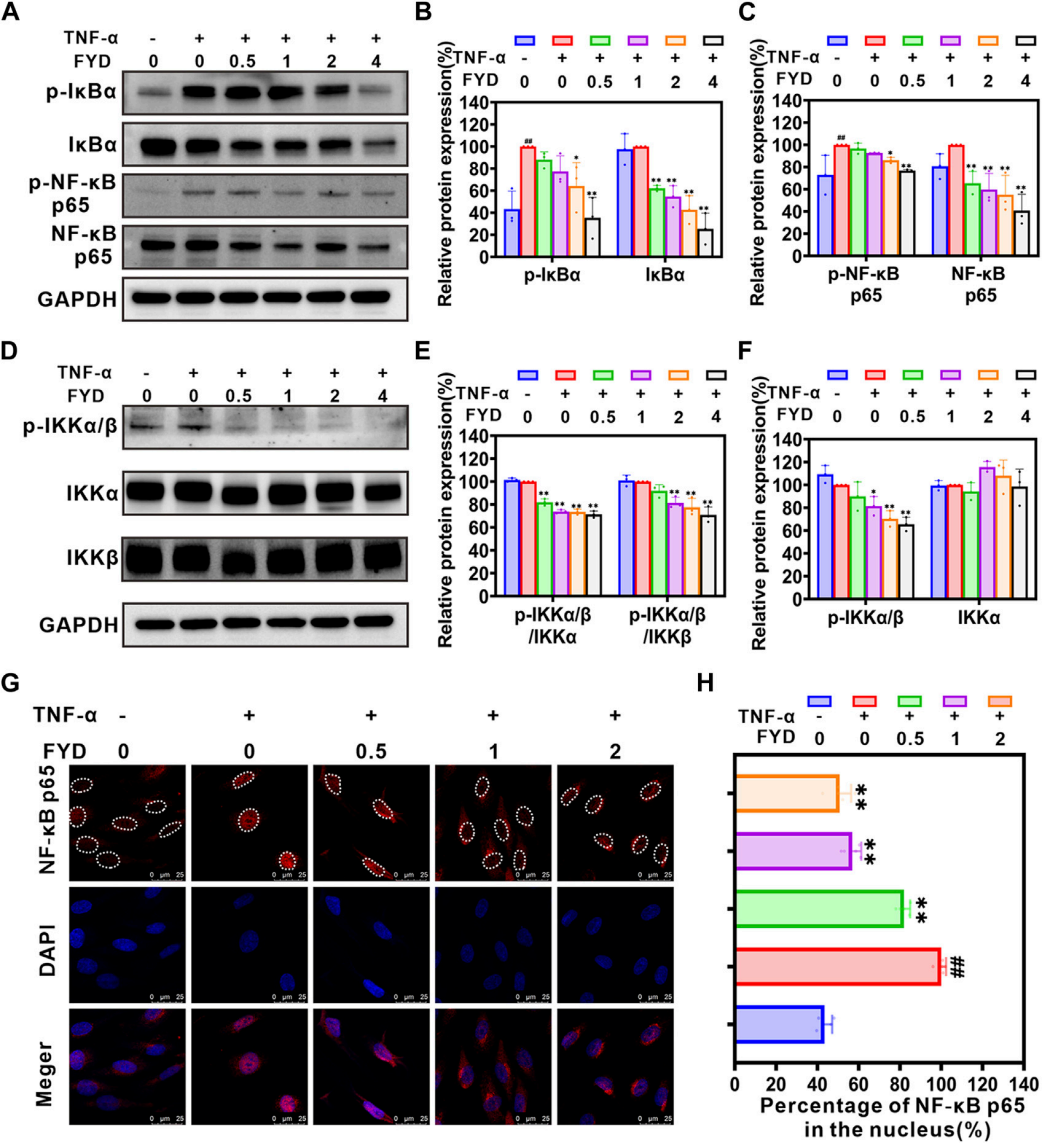
The expression of CAMs in HUVECs is downregulated by FYD through modulation of the NF-κB signaling pathway. **(A–F)** The HUVECs were pretreated with FYD at different concentrations (0, 0.5, 1.0, and 2.0 mg/mL) for a duration of 24 h followed by stimulation with TNF-α (10 ng/mL) for an additional 4 h. Western blot analysis was performed to assess the expression levels of IκBα, p-IκBα (Ser32), IKKα, IKKβ, p-IKKα/β (Ser176/180), NF-κB p65, and p-NF-κB p65 (Ser536). GAPDH was used as an internal control. Band intensity quantification was conducted using Image Lab software and expressed as a percentage relative to the control group treated with TNF-α alone. **(G)** The impact of FYD on the nuclear translocation of NF-κB p65 was assessed through immunofluorescence staining and visualized using a confocal microscope. NF-κB p65 was labeled with Alexa Fluor 594 (red), while the cell nucleus was labeled with DAPI (blue). **(H)** The nuclear/total fluorescence intensity ratio of NF-κB p65 (labeled with Alexa Fluor 594) was quantified using Fiji ImageJ software and expressed as a percentage of the control (treated with TNF-α alone). The data are presented as mean ± SD (*n* = 3–6); **indicate *p* < 0.01 compared to the Control (treated with TNF-α alone); ^##^ indicate *p* < 0.01 compared to the Negative control (without TNF-α).

The NF-κB signaling pathway has been extensively demonstrated to play a pivotal role in the regulation of expression of CAMs (Jayakumar et al., 2014; Zeng et al., 2021). Here, we employed western blot analysis to investigate the effect of FYD on the expression of CAMs in HUVECs via modulation of the NF-κB signaling pathway. As shown in Figures 3A–F, the NF-кB signaling pathway is activated by TNF-α through upregulation of NF-кB p65 expression and phosphorylation of p-IкBα (Ser 32), p-IKKα/β (Ser 176/180) and p-NF-кB p65 (Ser 536). Treatment with FYD in HUVECs resulted in a significant concentration-dependent reduction in the total protein level of IкBα and NF-кB p65, as well as the phosphorylation levels of p-IкBα (Ser 32), p-IKKα/β (Ser 176/180) and p-NF-кB p65 (Ser 536), compared to the TNF-α stimulated HUVECs. Additionally, the p-IKK/IKK ratio exhibited a significant reduction in comparison to HUVECs stimulated with TNF-α. We performed additional analysis to investigate the impact of FYD on NF-кB p65 nuclear translocation using immunofluorescence staining. The results revealed a significant dose-dependent inhibition of NF-кB p65 nuclear translocation in HUVECs by FYD (Figures 3G,H). These findings suggest that FYD exerts inhibitory effects on the NF-κB signaling pathway, leading to the degradation of NF-кB p65 through blockade of its nuclear translocation. Consequently, the downregulation of CAMs expression on HUVECs by FYD is closely associated with modulation of the NF-κB signaling pathway.

### 3.5 The migration and invasion of CNE1 cells are effectively suppressed by FYD

**FIGURE 4:**
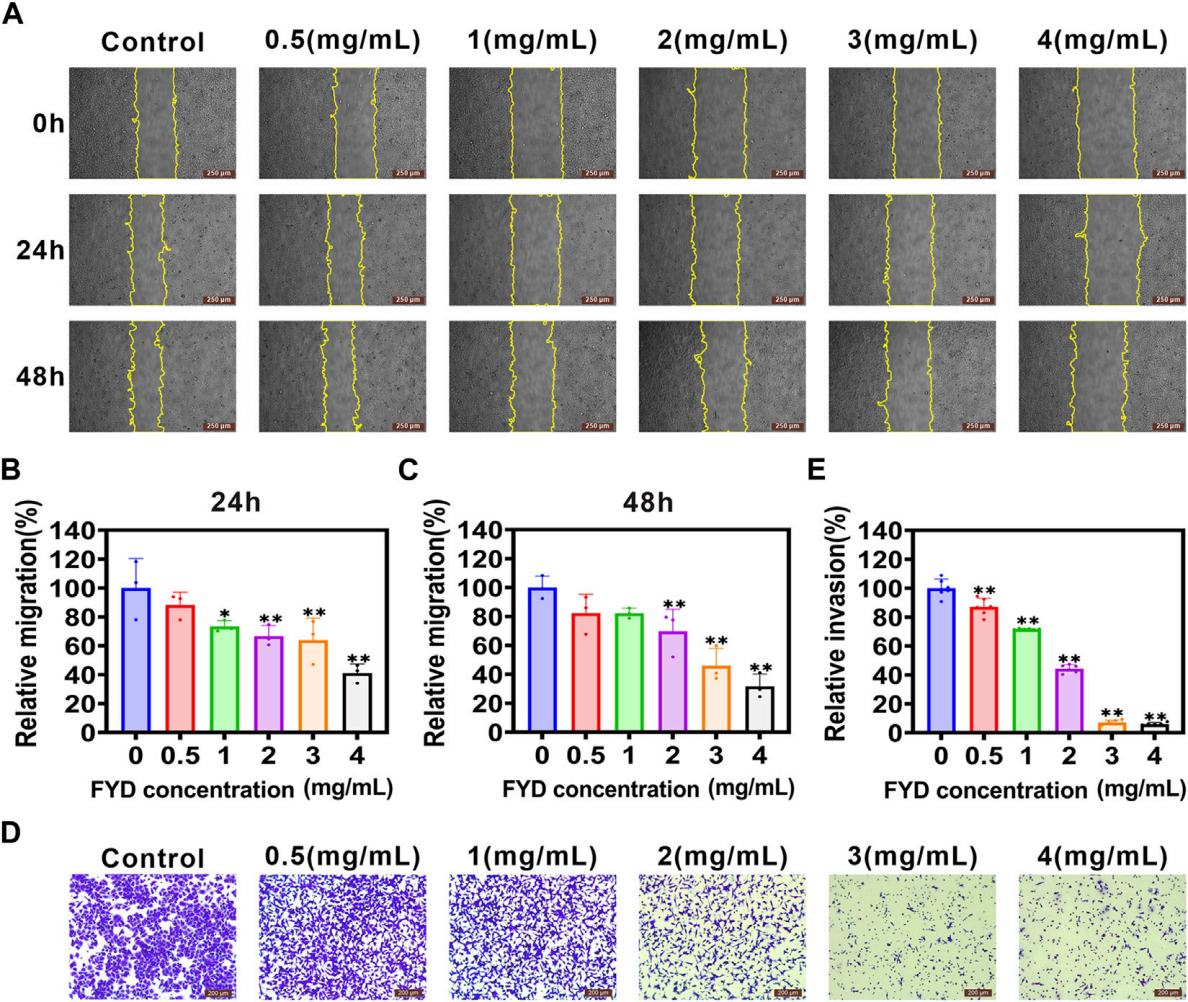
Repression of migration and invasion in CNE1 cells is observed upon FYD treatment. **(A)** The cell migration ability was assessed using a wound-healing assay in CNE1 cells treated with various concentrations of FYD for 24 and 48 h **(B, C)** The relative migration area was quantified using the Fiji ImageJ software and expressed as a percentage of control (FYD free). The regions of migration were delineated by yellow solid lines. **(D, E)** The cell invasion ability was assessed using the transwell assay in CNE1 cells treated with various concentrations of FYD for 24 h. The relative invasion rate was calculated as a percentage of the control group (without FYD treatment). The data are presented as mean ± SD (*n* = 3–6); **indicate *p* < 0.01 compared to the Control (without FYD treatment).

Another crucial step in the metastatic process involves the transmigration of CTCs across endothelial cells lining blood vessels, with cancer cell aggressiveness playing a pivotal role in this intricate mechanism (Wang et al., 2022). Here, we investigated the effect of FYD on the migratory and invasive behavior of CNE1 cells *in vitro*. The wound-healing assays revealed a concentration-dependent inhibition of CNE1 cell migration by FYD, with noticeable effects observed at concentrations of 3 mg/mL and 4 mg/mL after 48 h (Figures 4A–C). Furthermore, transwell assays demonstrated that FYD effectively suppressed CNE1 cell invasion in a concentration-dependent manner, with the group treated with 4 mg/mL showing only 6% invasion compared to the control group (Figures 4D,E). These results indicate that FYD exerts a significant inhibitory effect on the migration and invasion of CNE1 cells.

### 3.6 FYD suppresses cancer cell invasiveness by attenuating the EMT, PI3K/AKT, and FAK signaling cascades

**FIGURE 5:**
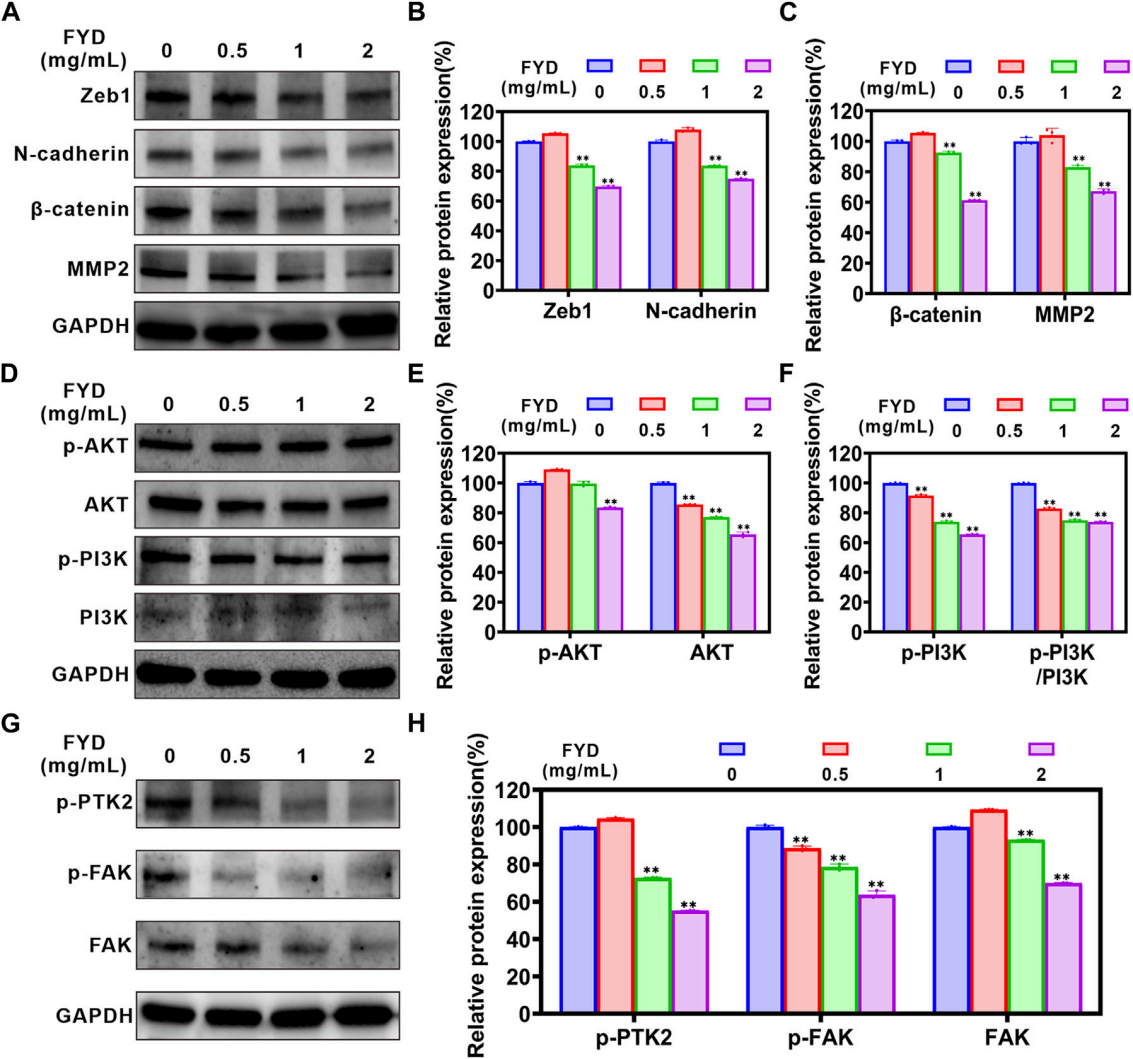
The effects of FYD on EMT, PI3K/AKT and FAK signaling pathways in CNE1 cells. Following a 24-h treatment with FYD, western blot analysis was performed to determine the expression levels of proteins associated with EMT, PI3K/AKT, and FAK pathways. GAPDH served as an internal control. Band intensity quantification was conducted using Image Lab software and expressed as a percentage relative to the control group (without FYD treatment). **(A–C)** Western blot analysis revealed that FYD effectively suppressed the expression of N-cadherin, β-catenin, ZEB1, and MMP2. **(D–F)** FYD dose-dependently inhibited both total and phosphorylation levels of PI3K and AKT proteins in CNE1 cells. **(G,H)** Dose-dependent treatment with FYD significantly attenuated the total and phosphorylation levels of FAK proteins in CNE cells. The data are presented as mean ± SD (*n* = 3); **indicate *p* < 0.01 compared to the Control (without FYD treatment).

The EMT process is strongly associated with cancer cell migration and invasion (Cheng et al., 2018; Liu et al., 2022). Therefore, we performed western blotting analysis to determine the effect of FYD on expression of EMT-related proteins in CNE1 cells. The results are shown in Figures 5A–C, FYD produced concentration-dependent inhibition of ZEB1, N-cadherin, β-catenin and MMP2 proteins expression, compared with the control group. We also performed qRT-PCR analysis to investigate the mRNA levels of EMT transcription-related factors, and found that the transcription levels of transcription factors ZEB1, FN1, and SNAIL2 was significantly reduced by FYD (Supplementary Figure S5).

The phosphatidylinositol-3-kinase (PI3K)/AKT signaling pathway participates in migration process of metastatic cancer cells, including the regulation of cytoskeleton-remodeling proteins and EMT–activating proteins that specifically regulate cell motility (Xue et al., 2012; Xue and Hemmings, 2013). We investigated the effect of FYD on the PI3K/AKT signaling pathway through western blot assay. The results demonstrated a significant concentration-dependent inhibition of p-PI3K (Tyr 458), AKT 1/2/3, and p-AKT 1/2/3 (AKT1-Tyr 315/AKT 2-Tyr 316/AKT 3-Tyr 312) expression by FYD, as compared to the control group (Figures 5D–F). Additionally, the p-PI3K/PI3K ratio exhibited a significant reduction. These findings suggest that FYD may inhibit the PI3K/AKT signaling pathway by inducing phosphorylation and total protein degradation. Moreover, the FAK signaling axis plays a pivotal role in facilitating cancer cell migration and invasion (Zhang et al., 2022). According to our findings, FYD exhibited a dose-dependent inhibition on the expression of FAK, p-FAK (Tyr 397), and p-PTK2 (Tyr 576/577) (Figures 5G,H). As a result of these findings, FYD suppresses the invasion and migration of cancer cell by regulating the EMT, PI3K/AKT, and FAK signaling cascades.

### 3.7 FYD effectively suppresses pulmonary metastasis

**FIGURE 6:**
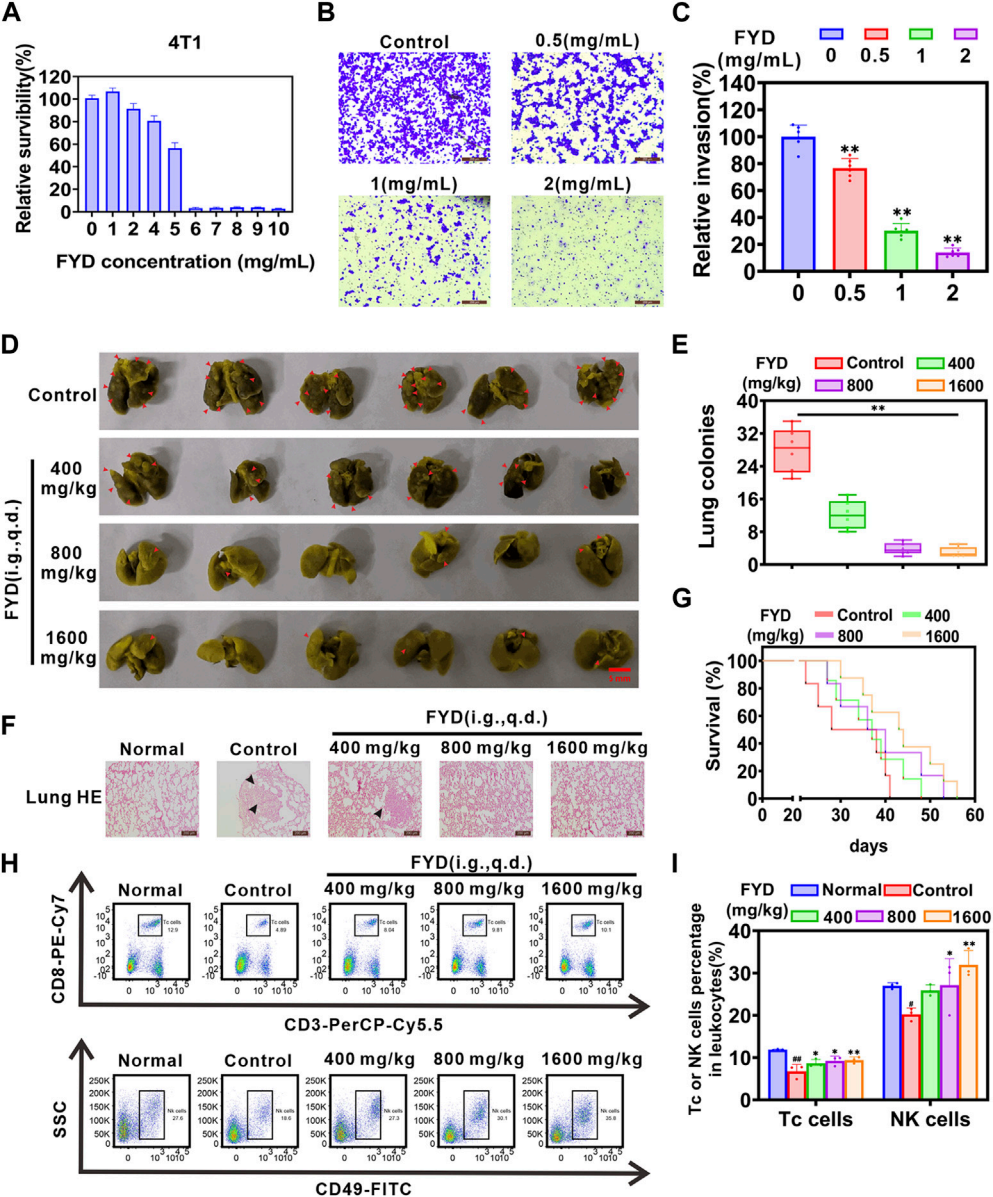
*In vivo* impact of FYD on cancer pulmonary metastasis in immunocompetent BALB/c mice. **(A)** The cytotoxicity of FYD to 4T1 cells were determined by CCK8 assay, and relative survibility was expressed as percentage of control. **(B)** The cell invasion ability was assessed using the transwell assay in 4T1 cells treated with various concentrations of FYD for 24 h. **(C)** The relative invasion rate was calculated as a percentage of the control group (without FYD treatment). **(D)** The formation of lung cancer nodules was observed *in vivo* in mice. Prior to inoculation with 4T1 cells (5 × 10^4^ cells/mouse, tail vein injection), the mice were pre-treated with FYD for 3 days at doses of 0, 400, 800, and 1600 mg/kg (100 μL volume per mouse, i.g., q.d.). The treatment was continued for a duration of 21 days. Representative images of the lungs displaying surface metastasis in mice are shown, with red arrows indicating the presence of breast cancer pulmonary nodules. **(E)** FYD significantly reduced the number of pulmonary metastatic nodules in a dose-dependent manner. **(F)** Representative images of lung sections stained with H&E from FYD-treated mice are shown. The black arrows indicate the area of breast cancer metastasis. **(G)** Evaluation of the survival period in mice treated with FYD. **(H)** The percentage of cytotoxic T lymphocytes (Tc cells, CD45^+^CD3^+^CD8^+^) and natural killer (NK) cells (CD45^+^CD49b+ cells) in peripheral blood of mice were assessed using flow cytometry. **(I)** Quantitative analysis revealed that FYD significantly elevated the proportions of Tc and NK cells in the peripheral blood, bringing them closer to the normal range. The data are presented as mean ± SD (*n* = 6); * indicate *p* < 0.05, ** indicate *p* < 0.01 compared to the Control (Pulmonary metastatic mice); # indicate *p* < 0.05, ## indicate *p* < 0.01 compared to the Normal (Healthy mice).

Due to the challenges in modeling nasopharyngeal carcinoma metastasis in immunocompetent syngeneic mice, the mechanisms of lung metastasis based on CTCs are similar for both breast cancer and nasopharyngeal cancer. In addition, *in vitro* CCK8 and transwell assays also showed that FYD could concentration-dependently inhibit the survival and invasion of mouse breast cancer 4T1 cells (Figures 6A–C), which was consistent with the results obtained in human nasopharyngeal carcinoma CNE1 cells (Figure 4D). Therefore, in this study, we utilized 4T1 breast cancer cells to examine the potential preventive effect of FYD on pulmonary metastasis *in vivo*. In this experiment, BALB/c mice were pretreated with FYD (0, 400, 800, and 1600 mg/kg) for a duration of 3 days, and then the mice received an intravenous injection of 4T1 cells via the tail vein. The doses administered to BALB/c mice were converted from clinical doses and *in vitro* 4T1 cell experiments (Bae et al., 2015; Nair et al., 2018). The administration of FYD continued to observe lung metastasis and survival rates. The results as shown in Figures 6D,E, revealed that FYD at doses of 800 mg/kg and 1600 mg/kg significantly reduced the formation of lung metastatic nodules induced by 4T1 cells. Histological hematoxylin and eosin (H&E) staining in Figure 6F demonstrated that the FYD-treated group had significantly fewer lung metastases compared to the control group. Moreover, FYD treatment extended the lifespan of mice with lung metastases, as depicted in Figure 6G. Immunohistochemical staining also showed that the expression of Ki67 was reduced in the lung metastasis tissue after FYD treatment, indicating that FYD treatment led to a weakened proliferation ability of 4T1 cells in lung tissue (Supplementary Figure S6). Furthermore, we further evaluated the effect of FYD for the PI3K/AKT and NF-κB signaling pathways in the lungs of mice through immunohistochemical staining. Interestingly, lung tumor metastases exhibited higher expression levels of PI3K, AKT and NF-κB p65 compared to healthy lung tissues; however, their expression significantly decreased following FYD treatment (Supplementary Figure S7). These findings suggest that FYD also effectively inhibits the PI3K/AKT and NF-κB signaling pathways *in vivo*. Importantly, no noticeable side effects were observed in the mice after 3 weeks of FYD treatment. The FYD treatment did not induce any significant changes in the body weight of the mice, while histological examination using H&E staining revealed that the FYD-treated mice exhibited normal morphological characteristics in their heart, liver, spleen, and kidney tissues (Supplementary Figure S8). The *in vivo* data revealed that FYD, even at weakly toxic doses, exhibits a remarkable inhibitory effect on the pulmonary metastasis.

It has been proposed that enhancing the immune system’s function and boosting immunity can help prevent cancer metastasis in the body (Lian et al., 2019). To investigate the effect of FYD on cellular immune function, flow cytometry was employed to measure the proportion of cytotoxic T lymphocytes (Tc cells) and natural killer cells (NK cells) in the peripheral blood. As shown in Figures 6H,I, FYD treatment significantly augmented the proportion of Tc cells (CD45^+^CD3^+^CD8^+^ cells) and NK cells (CD45+CD49b+ cells) in the peripheral blood, approaching physiological levels. These findings suggest that FYD reduces cancer metastasis and strengthens the anti-cancer immune response by promoting the release of Tc and NK cells by the immune system.

## 4 Discussion

**FIGURE 7:**
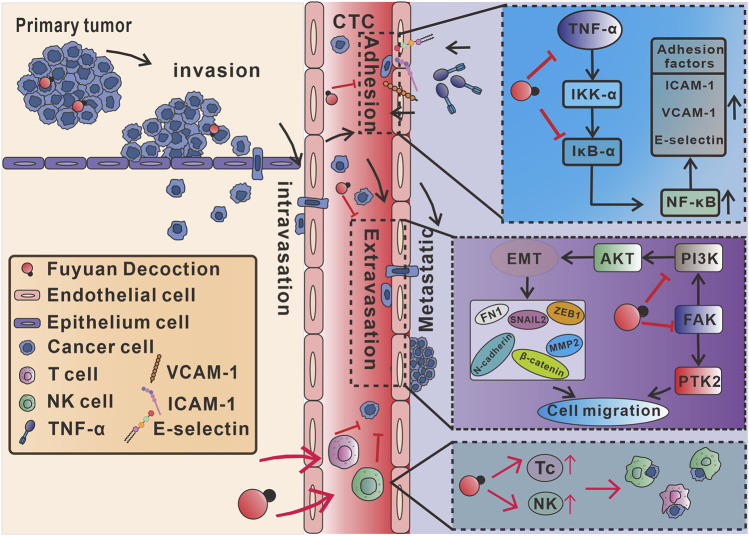
Mechanism of action of multifunctional FYD in preventing cancer metastasis. FYD interrupts adhesion of CTCs to vascular endothelium by suppressing CAMs through inhibition of the TNF-α-stimulated NF-кB signal pathways in endothelial cells. Additionally, FYD reduces motility of CTCs by regulating EMT, PI3K/AKT and FAK signaling pathways, thereby the inhibiting transendothelial migration (extravasation) of CTCs. Furthermore, FYD enhances anti-cancer immune responses by substantially increasing the subsets of Tc and NK cells in the peripheral immune system. The combined effects of FYD resulted in the continuous exposure of CTCs to the hostile microenvironment of the bloodstream, rendering them susceptible to death induced by anoikis, shear stress, and immune elimination.

The present studies confirmed for the first time that the FYD has a significant preventive effect on cancer metastasis due to its multifunctionality, and its effect is comprehensive and safe. At the molecular and cellular level, FYD inhibits CTCs adhesion to the vascular endothelial cells by down-regulating the expression of CAMs on the surface of endothelial cells induced by the inflammatory factor. Further mechanism studies showed that FYD reduced CAMs expression in endothelial cells by down-regulating the phosphorylation levels of IKK-α/β, IκB-α and NF-κB p65, as well as the nuclear translocation of NF-кB p65, thereby inhibiting the NF-κB signaling pathway activated by inflammatory factors. Furthermore, FYD inhibits cancer cells migration and invasion by inhibiting the EMT, PI3K/AKT, and FAK signal cascades. In addition, at the animal level, we also showed that FYD enhances anti-cancer immune responses by substantially increasing the subsets of Tc and NK cells in the peripheral immune system. A series of the beneficial effects of FYD collectively resulted in the failure of CTCs to adhere to the endothelium and transendothelial migration, prompting CTCs to continue to be exposed to the hostile bloodstream microenvironment and die under the attack of immune cells and the action of anoikis, thus effectively preventing cancer metastasis (Figure 7).

Our previous studies have demonstrated that inflammatory factors (including IL-1β, TNF-α and TNF-β, et al.) induce the expression of CAMs (such as ICAM-1, VCAM-1, and E-selectin) on the surface of endothelial cells by activating the NF-κB signaling pathway, which is crucial for CTCs to adhere to the vascular endothelial cells of the distant metastatic tissues (the most important and first step of metastatic cascade) (Lu et al., 2014; Lian et al., 2016; Wang et al., 2022). It is clear now that NF-κB plays an essential role as a dimeric transcription factor in the expression of CAMs activated by inflammatory cytokines (Hoesel and Schmid, 2013). In the resting state, NF-κB binds to its repressor protein IκB in the cytoplasm, blocking the entry transcription of NF-κB target genes into the nucleus. However, in the presence of inflammatory factors, IKK complex (IKKβ and IKKα) is activated and causes the phosphorylation and degradation of IκB protein, allowing NF-κB to enter the nucleus to initiate transcription of target genes (Perkins, 2006; Hayden and Ghosh, 2008). Because of the presence of at least one κB binding site in the gene promoters of ICAM-1, VCAM-1, and E-selectin, activation of the NF-κB signaling pathway rapidly promotes their expression (Lian et al., 2016). In the current study, we found that FYD significant interrupts adhesion of CNE1 cells to HUVECs by suppressing CAMs via inhibition of the NF-κB signaling pathway in endothelial cells (Figure 2; Figure 3). These encouraging results may be due to the fact that FYD contains a variety of anti-inflammatory compounds, including Fraxetin (Deng et al., 2022), Scutellarin (Wang et al., 2019; Peng et al., 2020; Mei et al., 2022), Ganoderic acid A (Jia et al., 2021; Zheng et al., 2022), Succinic acid (Krajka-Kuzniak et al., 2019), Ursolic acid (Yuan et al., 2021), Coumaric acid (Yoon et al., 2014), Rosmarinic acid (Han et al., 2017; Jin et al., 2021), Salvianolic acid B (Wang et al., 2022; Zhao et al., 2023), Luteolin (Aziz et al., 2018; Li et al., 2019) (Supplementary Table S2), which not only inhibit the NF-κB signaling pathway, but also inhibit the levels of inflammatory factors (e.g., IL-1β, IL-6, TNF-α). Therefore, the synergistic effect of multiple compounds prompted FYD to show a more significant anti-inflammatory effect compared with single agents.

Transendothelial migration is another critical process for successful metastasis of CTCs adhering to vascular endothelial cells, which requires CTCs to acquire enhanced motility (Wang et al., 2022). The motility of CTCs is tightly regulated by multiple signaling pathways, including EMT, PI3K/AKT, and FAK signaling pathways, and their regulatory mechanisms are extremely complex. The EMT process is mediated by transcription factors such as SNAIL, ZEB and Twist, and enhances CTCs motility through activation of mesenchymal markers (e.g., N-cadherin and β-catenin) (Dongre and Weinberg, 2019). Activation of the PI3K/AKT signaling pathway promotes CTCs to undergo EMT and accelerates the malignant progression of cancer metastasis (Xue and Hemmings, 2013; Li et al., 2022). Furthermore, FAK also plays a critical role in cytoskeletal organization, focal adhesion formation, and cell motility (Lietha et al., 2007; Zhang et al., 2022). Therefore, the invasion ability of CTCs may be effectively inhibited through multi-target co-regulation, and Chinese herbal compounds have unique advantages in this respect. In this study, we found that FYD inhibits cancer cells migration and invasion by inhibiting the EMT, PI3K/AKT, and FAK signal cascades (Figure 4; Figure 5), due to the fact that FYD contains multiple active components (including Scutellarin (Li et al., 2019; Wang et al., 2019), Ganoderic Acid A (Cheng and Xie, 2019), Fraxetin (Ma et al., 2022), Butein (Rampogu et al., 2021), et al.) that can act on these signaling pathways simultaneously.

## 5 Conclusion

In the present study, we demonstrated that FYD effectively prevents metastasis of nasopharyngeal carcinoma at non-cytotoxic concentrations by inhibiting the adhesion of CTCs to endothelial cells and their subsequent transendothelial migration, as well as enhancing anti-cancer immune response (Figure 7). Mechanistically, FYD blocks adhesion of CTCs to vascular endothelium by suppressing TNF-α-induced expression of CAMs through regulation of the NF-κB signaling pathway in endothelial cells. Moreover, FYD inhibits CTCs invasion and migration by attenuating EMT, PI3K/AKT and FAK signaling cascades. Additionally, FYD enhances the anti-cancer immune response by significantly augmenting the population of Tc and NK cells in the peripheral immune system. Unlike the low effectiveness and harmful side effects associated with standard chemotherapy medication treatment, FYD can safely and efficiently inhibit cancer metastasis at low-risk doses through multi-target and multifunctional properties. This new finding provides a modern medical basis for the application of FYD in the prevention of cancer metastasis, suggesting that multi-drug and multi-target synergistic therapy may be one of the most effective ways to prevent cancer metastasis.

## Data Availability

The original contributions presented in the study are publicly available. The data can be found here: https://pan.baidu.com/s/1VqV1p-GqE4XdAdTwECg9qw/ extraction code: 20nu.

## Abbreviations

FYD, Fuyuan Decoction; CTCs, Circulating tumor cells; NPC, Nasopharyngeal carcinoma; CAMs, Cell adhesion molecules; EMT, Epithelial-to-mesenchymal transition; TCM, Traditional Chinese medicine; PBS, Phosphate buffer saline; FBS, Fetal bovine serum; ECM, Endothelial cell culture medium; ECGS, endothelial cell growth supplement; CCK8, cell counting kit 8; PI, Propidium iodide; SDS-PAGE, sodium dodecyl sulfate polyacrylamide gel; H&E, Hematoxylin and eosin; NK, Natural killer.
