# Supplementary material for: Fuyuan decoction prevents nasopharyngeal carcinoma metastasis by inhibiting circulating tumor cells/ endothelial cells interplay and enhancing anti-cancer immune response
Source: Front Pharmacol. 2024 Apr 25;15:1355650. doi: 10.3389/fphar.2024.1355650 (PMC11084272; doi:10.3389/fphar.2024.1355650)
Supplement: Supplementary file 1 [file DataSheet1.docx]

**Supplementary** **Materials and Methods**

**Compound identification of FYD**

The chemical composition of FYD was determined using the UPLC-HRMS methodand compared with secondary spectra in the mass spectrometry database to confirm the results. FYD lyophilized powder was dissolved in 70% methanol aqueous solution to a concentration of 50 mg/mL. The solution was then sonicated for 30 min and centrifuged at 16000 g for 15 minutes at 4 °C. The supernatant was collected and vacuum freeze dried. The lyophilized powder was then redissolved in 40% methanol aqueous solution, centrifuged, and collected the supernatant. The test solution (FYD) was precisely aspirated and analyzed by LC-MS. The UPLC conditions were as follows: chromatography was performed on an ACQUITY UPLC HSS T3 (2.1 × 100 mm, 1.8 µm) column, mobile phase consisted of 0.1% formic acid aqueous solution (A) and 0.1% formic acid acetonitrile solution (B), the flow rate was kept at 0.3 mL/min and the column temperature was maintained at 35 °C. Gradient elution of the mobile phase was 95% A and 5% B from 0 to 17.0 min, 2% A and 98% B from 17.0 to 17.2 min, and 95% A and 5% B from 17.2 to 20.0 min. The parameters of the Thermo Q-Exactive HFX mass spectrometry were set as follows: mass spectrometry analysis was conducted in positive and negative ion modes, spray voltage of positive ion mode was 3.8 kV, spray voltage of negative ion mode was 3.0 kV, sheath gas flow rate was 45 arbitrary units (arb), aux gas flow rate was 20 arb, aux gas heater temperature was 370 °C, capillary temperature was 320 °C.

**Cell culture**

Human nasopharyngeal carcinoma cells CNE1 and mouse breast cancer cells 4T1 were obtained from Cell Bank of Chinese Academy (Shanghai, China). Human nasal epithelial cells HNEPC were obtained from Otwo Biotech (ShenZhen, China) Inc. CNE1 cells cultured in Roswell Park Memorial Institute (RPMI) 1640 medium (Hyclone) supplemented with 10% fetal bovine serum (FBS, Gibico), 100 U/mL penicillin, and 100 μg/mL streptomycin. 4T1 and HNEPC cells were cultured in Dulbecco’s modified Eagle’s medium (DMEM) high-glucose medium (Hyclone) supplemented with 10% fetal bovine serum (FBS, Gibico), 100 U/mL penicillin, and 100 μg/mL streptomycin. HUVECs were extracted from human umbilical veins and grown in endothelial cell culture medium (ECM, ScienCell) with 5% FBS and 100 μg/mL endothelial cell growth supplement (ECGS, ScienCell), 100 U/mL penicillin, and 100 μg/mL streptomycin. All cells were incubated in a 5% CO_2_ incubator at 37 °C and harvested with 0.25% trypsin (GenView).

**Cell viability assay**

The cytotoxicity of FYD was measured using the cell counting kit 8 (CCK8) as our previously described methods (Wang, W. et al., 2022; Zhong et al., 2020). Cells (1 × 10^4^/well) were seeded in 96-well plates and cultured for 24 h. The cells were then treated with various concentrations of FYD (0, 1, 2, 4, 5, 6, 7, 8, 9, and 10 mg/mL) for 24 h. After that, 10 μL of CCK8 was added and co-incubation for 1 h. Cell viability was assessed by measuring absorbance at 450 nm in a multifunctional microplate reader (TECAN, M200 PRO), and the results were presented as a relative ratio compared to the untreated control.

**Cell cycle assay**

The impact of FYD on the cell cycle was evaluated using the cell cycle test kit (Lu et al., 2014). The cells were seeded in a 6-well plate (1 × 10^5^ cells per well) for 24 h. The cells were then treated with various concentrations of FYD (0, 0.5, 1, 2, 3, and 4 mg/mL) for 24 h, washed twice with PBS, and fixed overnight at 4°C with 75% ethanol. After that, the cells were washed with PBS and stained with propidium iodide (PI) staining working solution for 45 min at room temperature in the dark. The cell cycle was detected by ﬂow cytometry (BD, FACSAriaIII) and analyzed using FlowJo software.

**Cell apoptosis assay**

Cell apoptosis was measured using the annexin V-FITC/PI staining kit (Cheng et al., 2018). The cells were seeded in a 6-well plate (1 × 10^5^ cells per well) for 24 h. After treatment with various concentrations of FYD (0, 0.5, 1, 2, 3, and 4 mg/mL) for 24 h, the cells were washed with PBS, resuspended in binding buffer, and stained with Annexin V-FITC and PI for 15 min in the dark. The cell apoptosis was assessed by ﬂow cytometry and analyzed using FlowJo software.

**Hetero-adhesion analysis of cancer cells to endothelial cells**

As previously mentioned methods, the effects of FYD on the heterogeneous adherence of CNE1 cells to HUVECs were investigated (Lian et al., 2016; Lu et al., 2019). cultured HUVECS were cultured in a 24-well plate and pretreated with FYD (0, 0.5, 1, 2, 4 mg/mL) for 24h, followed by stimulation with TNF-α (10 ng/mL) for 4 h. FYD and Rhodamine 123-labeled CNE1 cells were then added to the HUVECs monolayers and co-cultured for 1 h. Non-adherent CNE1 cells were removed and washed with PBS. The adhering CNE1 cells (labeled with Rhodamine 123) on endothelial cells were photographed using a ﬂuorescence microscope (Leica, Germany). A comparison of the number of adhered cancer cells in the treated group and control group was used to calculate the inhibition of adhesion.

**Effect of FYD on the expression of cell adhesion molecules on HUVECs**

The effect of FYD on the expression of CAMs (including ICAM-1, VCAM-1, and E-selectin) on HUVECs was analyzed by ﬂow cytometry (Lu et al., 2014; Wang, W. et al., 2022). HUVECs were seededin a 6-well plate (1 × 10^5^ cells per well) for 24 h. Then the HUVECs were pretreated with various concentrations of FYD (0, 0.5, 1, 2, and 4 mg/mL) for 24 h, followed by stimulation with TNF-α (10 ng/mL) for 4 h. HUVEC were digested, resuspended, and stained with CD106 (PE-labeled), CD54 (FITC-labeled), and CD62E (APC-labeled) antibodies, respectively. The isotype-matched control antibody was used to test the background staining. The expression of E-selectin, ICAM-1, and VCAM-1 on HUVECs surface was detected by flow cytometry and analyzed using FlowJo software.

**Effect of FYD on NF-κB signaling pathway on HUVECs**

To examine the effect of FYD on the TNF-α stimulated NF-κB signaling pathway, HUVECs were seeded in a 6-well plate (1 × 10^5^ cells per well) for 24 h. The HUVECs were pretreated with various concentrations of FYD (0, 0.5, 1, 2, and 4 mg/mL) for 24 h, followed by stimulation with TNF-α (10 ng/mL) for 4 h. After incubation, cells were lysed, and the protein expression levels of IκBα, p-IκBα (Ser 32), IKKα, IKKβ, p-IKKα/β (Ser 176/180), NF-κB p65, and p-NF-κB p65 (Ser 536) were tested by western blot analysis. The nuclear translocation of NF-κB p65 was analyzed by immunoﬂuorescence staining and photographed using a confocal microscope (Wang, W. et al., 2022).

**Migration assay**

As previously reported methods, the effect of FYD on CNE1 cell migration was measured using the wound-healing migration assay (Cheng et al., 2018; Wang, W. et al., 2022; Zhong et al., 2020). CNE1 cells were seeded in a 24-well plate. After the formation of cell monolayers, the cells were scratched, washed thrice with PBS, and grown in serum-free medium containing various doses of FYD (0, 0.5, 1, 2, 3, and 4 mg/mL). The same wounded areas were imaged at different time points (0, 24, and 48 h) and quantiﬁed using Fiji Image J software. A comparison of the initial area and the ﬁnal area of the marked areas were used to evaluate the cell migration ability.

**Invasion assay**

The effect of FYD on the invasion ability of CNE1 cells was evaluated using transwell invasion assay. Transwell culture chambers (24-well, 8 μm pore size, Costar, Corning, USA) were placed in 24-well plates containing 500 μL of medium supplemented with 20% FBS and different concentrations of FYD (0, 0.5, 1, 2, 3, and 4 mg/mL). The 5×10^4^ CNE1 cells were suspended in 200 μL of serum-free medium containing same concentration of FYD as above were seeded in the upper chamber of the wells. After another 24 h of incubation at 37°C in 5% CO_2_, the cells in the upper chambers were carefully cleaned with a cotton swab, washed with PBS, and the upper chambers basolateral side were fixed with 4% (w/v) paraformaldehyde and stained with crystal violet. The invading cells were counted and photographed using an optical microscope (Leica, Germany) (Wang, W. et al., 2022).

**Effect of FYD on Epithelial-to-mesenchymal transition, PI3K/AKT and FAK/Paxillin signaling pathways on cancer cells**

CNE1 cells were seeded in 6-well plate (1 × 10^5^ cells per well). After incubation for 24 h. The cell medium was replaced into fresh media with different concentrations of FYD (0, 0.5, 1, and 2 mg/mL) for another 24 h. The cells were lysed and the protein expression levels of Zeb1, MMP2, N-cadherin, β-catenin, AKT1/2/3, p-AKT1/2/3 (AKT1-Tyr 315/AKT2-Tyr316/AKT3-Tyr312), PI3K, p-PI3K (Tyr 458), FAK, p-FAK (Tyr 397) and p-PTK2 (Tyr 576/577) were tested by western blot analysis. The mRNA expression levels of SNAIL2, ZEB1, FN1, and 18s rRNA were analyzed by quantitative reverse transcription-quantitative polymerase chain reaction (qRT-PCR).

**Western blot analysis**

Western blot analysis was performed as previously described methods (Cheng et al., 2018; Lu et al., 2014; Wang, W. et al., 2022). The cells were washed twice with PBS and lysed on ice for 30 min with RIPA lysate (Pierce) containing 1% phosphatase inhibitor and 1% PMSF. After the centrifugation at 12,000 g for 20 min at 4 °C, the supernatant was saved for western blot analysis. The BCA protein assay kit was used to test protein concentrations. The protein samples were denatured with loading buffer containing sodium dodecyl sulfate (SDS) at 100 °C for 10 min. Equal quantities of denatured protein samples were separated on a 4%-20% (w/v) sodium dodecyl sulfate polyacrylamide gel (SDS-PAGE) and then transferred to PVDF membranes (Bio-Rad). After washing with TBST, blocking with 5% BSA for 1.5 h at room temperature, and incubating overnight at 4°C with primary antibodies (1:2,000 dilution) , the membranes were washed with TBST and incubated with HRP-conjugated secondary antibodies (1:10,000 dilution) for 2 h at room temperature. The membranes were then washed with TBST and exposed to the ChemiDoc XRS System (Bio-Rad) to detect the expressions of the target proteins. The ECL Kit (Pierce) was used to visualize band signals, and the Image Lab software (Bio-Rad) was used to quantify band intensities.

**RNA extraction and qRT-PCR assay**

Total RNA was isolated using TRIzol reagent (Life Technologyies) according to manufacturer’s protocol. The RNA concentrations were measured using the BioDrop (BioDrop, United Kingdom), and mRNA was reverse transcribed into cDNA using the TransScriptR All-in-One First-Strand cDNA Synthesis SuperMix (Transgen). The quantitative PCR (qPCR) was performed using the CFX96 touch real-time PCR detection system (iQ5, Bio-Rad) with PerfectStart™ Green qPCR SuperMix (Transgen) under thermocycling conditions( initial denaturation at 95°C for 30 sec; followed by 40 cycles of 95°C (5 s) and 60°C (30 s)) (Wang, W. et al., 2022). The 18s rRNA was used as a normalization control and the primer sequences for qPCR were presented in **Supplementary Table S1**.

**In vivo pulmonary metastasis assay**

The effect of FYD on the metastasis of 4T1 breast cancer in BALB/c mice was investigated. Experimental metastasis was performed as previously described methods (Lu et al., 2019; Wang, W. et al., 2022; Zhong et al., 2020). Mice were randomly divided into six groups (n= 16 in each group): (control group, model group, 0 mg/kg FYD group, 400 mg/kg FYD group, 800 mg/kg FYD group, and 1600 mg/kg FYD group). The 6 mice in each group were used to observe mouse tumor metastasis, and other 10 mice were examined for survival rate. After digestion with 0.05% trypsin-EDTA and washed twice with PBS, 4T1 cells were resuspended and adjusted the cell density to 5 × 10^5^ cells/mL. The female BALB/c mice were injected with 100 μl of 4 T1 cells (5 × 10^4^ cells/mouse) through the tail vein. Mice were pre-treated with FYD for 3 days [0, 400, 800 and 1600 mg/kg, FYD lyophilized powder was dissolved in ultrapure water at a concentration of 100 μl per mouse, i.g. (gavage), q.d (quaque die)] prior to inoculation with 4T1 cells, followed by continued treatment for 21 days. The mice were sacrified to collect tissue samples. The lungs were preserved with Bouin's solution and the lung tumor nodules were counted. For histopathological examination, the heart, liver, spleen, and kidney tissues of mice were fixed with formalin, embedded in paraffin, and stained with hematoxylin and eosin (H&E). Flow cytometry analysis was used to count the amount of cytotoxic T lymphocytes (Tc cells) and natural killer cells (NK cells) in the blood and spleen. The mouse survival curve analysis (ten mice for each group) was performed using Prism 7.0 software.

**References**

Cheng Y, Lu Y, Zhang D, Lian S, Liang H, Ye Y, Xie R, Li S, Chen J, Xue X, et al. 2018. Metastatic cancer cells compensate for low energy supplies in hostile microenvironments with bioenergetic adaptation and metabolic reprogramming. Int J Oncol. Dec;53:2590-2604. Epub 2018/10/04.

Lian S, Lu Y, Cheng Y, Yu T, Xie X, Liang H, Ye Y, Jia L. 2016. S-nitrosocaptopril interrupts adhesion of cancer cells to vascular endothelium by suppressing cell adhesion molecules via inhibition of the NF-small ka, CyrillicB and JAK/STAT signal pathways in endothelial cells. Eur J Pharmacol. Nov 15;791:62-71. Epub 2016/10/30.

Lu Y, Lian S, Ye Y, Yu T, Liang H, Cheng Y, Xie J, Zhu Y, Xie X, Yu S, et al. 2019. S-Nitrosocaptopril prevents cancer metastasis in vivo by creating the hostile bloodstream microenvironment against circulating tumor cells. Pharmacol Res. Jan;139:535-549. Epub 2018/10/27.

Lu Y, Yu T, Liang H, Wang J, Xie J, Shao J, Gao Y, Yu S, Chen S, Wang L, et al. 2014. Nitric oxide inhibits hetero-adhesion of cancer cells to endothelial cells: restraining circulating tumor cells from initiating metastatic cascade. Sci Rep. Mar 11;4:4344. Epub 2014/03/13.

Wang D, Li Q, Liu R, Xu H, Yin Y, Wang Y, Wang H, Bi K. 2019. Quality control of Semen Ziziphi Spinosae standard decoction based on determination of multi-components using TOF-MS/MS and UPLC-PDA technology. J Pharm Anal. Dec;9:406-413. Epub 2020/01/01.

Wang W, Fu C, Lin M, Lu Y, Lian S, Xie X, Zhou G, Li W, Zhang Y, Jia L, et al. 2022. Fucoxanthin prevents breast cancer metastasis by interrupting circulating tumor cells adhesion and transendothelial migration. Front Pharmacol.13:960375. Epub 2022/09/27.

Zhong C, Yang J, Lu Y, Xie H, Zhai S, Zhang C, Luo Z, Chen X, Fang X, Jia L. 2020. Achyranthes bidentata polysaccharide can safely prevent NSCLC metastasis via targeting EGFR and EMT. Signal Transduct Target Ther. Aug 31;5:178. Epub 2020/09/02.

**Supplementary Tables**

**Supplementary Table 1.** Primer sequences used for reverse transcription-quantitative polymerase chain reaction analyses of human nasopharyngeal carcinoma cells CNE1.

| **Gene** | **Primer sequence (5’-3’)** | |
| --- | --- | --- |
| SNAIL2 | | F: GATGCCGCGCTCCTTCCTGG |
|  |  | R: TGGAGCAGCGGTAGTCCACAC |
| ZEB1 | | F: ATCCTGGGGCCTGAAGCTCAGG |
|  |  | R: TGGTGTGCCCTGCCTCTGGT |
| FN1 | | F: TGCAAGGCCTCAGACCGGGT |
|  |  | R: GCGCTCAGGCTTGTGGGTGT |
| 18s | | F: AGAAACGGCTACCACATCCA |
|  |  | R: CCCTCCAATGGATCCTCGTT |

FN, Fibronectin; SNAIL, snail family transcriptional repressor; ZEB, Zinc-finger E-box-binding homeobox.

**Supplementary Table 2.** Compound type and quantity.

| **subclass** | **Freq** |
| --- | --- |
| (3_>5)_dinucleotides | 1 |
| other | 8 |
| other | 1 |
| Anthracenecarboxylic_acids_and_derivatives | 3 |
| Anthraquinones | 9 |
| other | 2 |
| other | 1 |
| Auronols | 1 |
| other | 1 |
| other | 2 |
| other | 1 |
| Oxadiazoles | 1 |
| Oxazoles | 1 |
| Pyrazoles | 2 |
| Imidazolidines | 1 |
| Pyrazolines | 1 |
| 2_phenoxypropionic_acids | 1 |
| Anilides | 8 |
| Benzenesulfonamides | 4 |
| Benzenesulfonic_acids_and_derivatives | 3 |
| Benzoic_acids_and_derivatives | 42 |
| Benzonitriles | 1 |
| Benzophenones | 3 |
| Benzoyl_derivatives | 1 |
| Benzyloxycarbonyls | 1 |
| Biphenyls_and_derivatives | 2 |
| Diphenylmethanes | 3 |
| N_phenylureas | 1 |
| Nitrobenzenes | 2 |
| other | 3 |
| Phenethylamines | 1 |
| Phenoxy_compounds | 1 |
| Phenoxyacetic_acid_derivatives | 3 |
| Phenyl_beta_methoxyacrylates | 2 |
| Phenylacetamides | 1 |
| Phenylmethylamines | 3 |
| Phenylpropanes | 2 |
| Phenylpropylamines | 1 |
| Styrenes | 2 |
| Sulfanilides | 2 |
| Terphenyls | 1 |
| Toluenes | 3 |
| Trifluoromethylbenzenes | 1 |
| other | 3 |
| Phenylbenzimidazoles | 2 |
| other | 1 |
| other | 3 |
| Benzofuranones | 3 |
| Dibenzofurans | 1 |
| 1_benzopyrans | 12 |
| 2_benzopyrans | 3 |
| other | 1 |
| Phenothiazines | 1 |
| other | 1 |
| Benzoxazinones | 3 |
| Carboximidic_acids | 2 |
| Amino_acids_peptides_and_analogues | 206 |
| Carboxylic_acid_derivatives | 2 |
| Carboxylic_acids | 4 |
| Dicarboxylic_acids_and_derivatives | 10 |
| Tetracarboxylic_acids_and_derivatives | 2 |
| Tricarboxylic_acids_and_derivatives | 7 |
| Cinnamic_acids | 1 |
| Hydroxycinnamic_acids_and_derivatives | 34 |
| other | 1 |
| Coumarin_glycosides | 6 |
| Furanocoumarins | 3 |
| Hydroxycoumarins | 13 |
| other | 3 |
| Pyranocoumarins | 2 |
| other | 1 |
| Chaetoglobosins | 2 |
| other | 3 |
| Linear_diarylheptanoids | 6 |
| Benzodiazines | 5 |
| Piperazines | 3 |
| Pyrimidines_and_pyrimidine_derivatives | 4 |
| Dibenzylbutanediol_lignans | 2 |
| other | 1 |
| Furanones | 7 |
| Oxirane_carboxylic_acids_and_derivatives | 1 |
| Fatty_acid_esters | 6 |
| Fatty_acids_and_conjugates | 61 |
| Fatty_acyl_glycosides | 27 |
| Fatty_alcohols | 5 |
| Fatty_amides | 7 |
| Lineolic_acids_and_derivatives | 23 |
| other | 1 |
| Flavans | 8 |
| Flavones | 16 |
| Flavonoid_glycosides | 59 |
| Hydroxyflavonoids | 1 |
| O_methylated_flavonoids | 29 |
| other | 4 |
| other | 1 |
| other | 3 |
| Tetrahydrofuran_lignans | 6 |
| Furoic_acid_and_derivatives | 2 |
| Glycosylglycerols | 2 |
| Glycerophosphocholines | 6 |
| Glycerophosphoethanolamines | 1 |
| other | 2 |
| Alpha_hydroxy_acids_and_derivatives | 2 |
| Beta_hydroxy_acids_and_derivatives | 4 |
| Medium_chain_hydroxy_acids_and_derivatives | 10 |
| Short_chain_hydroxy_acids_and_derivatives | 1 |
| Purines_and_purine_derivatives | 11 |
| Carbazoles | 1 |
| Indolecarboxylic_acids_and_derivatives | 2 |
| Indoles | 5 |
| Indolines | 1 |
| Indolyl_carboxylic_acids_and_derivatives | 6 |
| N_alkylindoles | 1 |
| other | 1 |
| Pyridoindoles | 2 |
| Pyrroloindoles | 1 |
| other | 1 |
| Isobenzofuranones | 1 |
| other | 1 |
| Coumestans | 2 |
| Furanoisoflavonoids | 2 |
| Hydroxyisoflavonoids | 1 |
| Isoflav_2_enes | 4 |
| Isoflavonoid_C_glycosides | 1 |
| Isoflavonoid_O_glycosides | 16 |
| O_methylated_isoflavonoids | 8 |
| Pyranoisoflavonoids | 1 |
| Isoindolines | 3 |
| other | 1 |
| Gamma_keto_acids_and_derivatives | 1 |
| Medium_chain_keto_acids_and_derivatives | 5 |
| Beta_lactams | 1 |
| Delta_valerolactones | 2 |
| Gamma_butyrolactones | 2 |
| other | 7 |
| Chalcones_and_dihydrochalcones | 11 |
| Cinnamylphenols | 1 |
| other | 1 |
| other | 1 |
| other | 2 |
| Zearalenones | 1 |
| other | 3 |
| Naphthalenecarboxylic_acids_and_derivatives | 4 |
| Phenylnaphthalenes | 2 |
| other | 3 |
| Naphthopyranones | 1 |
| other | 1 |
| 3_5_cyclic_purine_nucleoside_phosphorothioates | 1 |
| other | 1 |
| other | 1 |
| Organic_phosphonic_acids | 1 |
| Organosulfonic_acids_and_derivatives | 1 |
| Arylsulfates | 1 |
| Sulfuric_acid_esters | 1 |
| Amines | 7 |
| Aminoxides | 1 |
| Guanidines | 1 |
| N_arylamides | 1 |
| Quaternary_ammonium_salts | 4 |
| Alcohols_and_polyols | 22 |
| Carbohydrates_and_carbohydrate_conjugates | 85 |
| Carbonyl_compounds | 27 |
| Ethers | 2 |
| Morpholines | 3 |
| other | 1 |
| other | 1 |
| Hybrid_peptides | 4 |
| other | 1 |
| Anisoles | 1 |
| other | 5 |
| 1_hydroxy_2_unsubstituted_benzenoids | 4 |
| 1_hydroxy_4_unsubstituted_benzenoids | 2 |
| Benzenediols | 7 |
| Methoxyphenols | 17 |
| Nitrophenols | 1 |
| Tyrosols_and_derivatives | 1 |
| other | 8 |
| Benzylpiperidines | 2 |
| Piperidinecarboxylic_acids_and_derivatives | 4 |
| Diterpenoids | 26 |
| Hopanoids | 1 |
| Monoterpenoids | 8 |
| Quinone_and_hydroquinone_lipids | 2 |
| Sesquiterpenoids | 19 |
| Sesterterpenoids | 1 |
| Terpene_glycosides | 57 |
| Terpene_lactones | 13 |
| Triterpenoids | 34 |
| other | 6 |
| Alloxazines_and_isoalloxazines | 2 |
| other | 9 |
| Purine_2_deoxyribonucleosides | 1 |
| Purine_3_deoxyribonucleosides | 1 |
| Cyclic_purine_nucleotides | 4 |
| Purine_nucleotide_sugars | 1 |
| Purine_ribonucleotides | 2 |
| Pyranones_and_derivatives | 5 |
| other | 1 |
| Halopyridines | 1 |
| Hydropyridines | 2 |
| Hydroxypyridines | 2 |
| other | 1 |
| Pyridine_carboxaldehydes | 1 |
| Pyridinecarboxylic_acids_and_derivatives | 9 |
| Pyridinium_derivatives | 1 |
| Pyridoxines | 2 |
| Pyrrolidinylpyridines | 1 |
| other | 2 |
| Pyrimidine_2_deoxyribonucleosides | 1 |
| Pyrimidine_2'_deoxyribonucleosides | 1 |
| Pyrrole_carboxylic_acids_and_derivatives | 1 |
| Substituted_pyrroles | 2 |
| Phenylpyrrolidines | 1 |
| other | 1 |
| Aminoquinolines_and_derivatives | 1 |
| Benzoquinolines | 1 |
| Nitroquinolines_and_derivatives | 1 |
| Phenylquinolines | 1 |
| Quinoline_carboxylic_acids | 2 |
| Quinolones_and_derivatives | 2 |
| other | 1 |
| other | 10 |
| Stemoamide_type_alkaloids | 1 |
| Androstane_steroids | 2 |
| Bile_acids_alcohols_and_derivatives | 2 |
| Cholestane_steroids | 1 |
| Cucurbitacins | 5 |
| Estrane_steroids | 4 |
| Hydroxysteroids | 4 |
| Oxosteroids | 3 |
| Pregnane_steroids | 2 |
| Steroid_esters | 1 |
| Steroid_lactones | 11 |
| Steroidal_glycosides | 8 |
| other | 4 |
| Stilbene_glycosides | 2 |
| Hydrolyzable_tannins | 4 |
| other | 1 |
| other | 1 |
| other | 2 |
| Bilirubins | 1 |
| Porphyrins | 1 |
| 1_3_5_triazines | 1 |
| other | 1 |
|  | 1430 |

**Supplementary Table 3.** The number of the top 6 compound classes and their subclasses.

| **class1** | **subclass** | **Freq** | **class_ID** | **subclass_ID** |
| --- | --- | --- | --- | --- |
| Benzene_and_substituted_derivatives | Anilides | 8 | Class_1(98) | Sub_2 |
|  | Benzoic_acids_and_derivatives | 42 | Class_1(98) | Sub_1 |
|  | other | 48 | Class_1(98) | Other |
| Carboxylic_acids_and_derivatives | Amino_acids_peptides_and_analogues | 206 | Class_2(231) | Sub_1 |
|  | Dicarboxylic_acids_and_derivatives | 10 | Class_2(231) | Sub_2 |
|  | other | 15 | Class_2(231) | Other |
| Fatty_Acyls | Fatty_acids_and_conjugates | 61 | Class_3(129) | Sub_1 |
|  | Fatty_acyl_glycosides | 27 | Class_3(129) | Sub_2 |
|  | other | 41 | Class_3(129) | Other |
| Flavonoids | Flavonoid_glycosides | 59 | Class_4(113) | Sub_1 |
|  | O_methylated_flavonoids | 29 | Class_4(113) | Sub_2 |
|  | other | 25 | Class_4(113) | Other |
| Organooxygen_compounds | Carbohydrates_and_carbohydrate_conjugates | 85 | Class_5(136) | Sub_1 |
|  | Carbonyl_compounds | 27 | Class_5(136) | Sub_2 |
|  | other | 24 | Class_5(136) | Other |
| Prenol_lipids | other | 70 | Class_6(161) | Other |
|  | Terpene_glycosides | 57 | Class_6(161) | Sub_1 |
|  | Triterpenoids | 34 | Class_6(161) | Sub_2 |

**Supplementary Table 4.** Results of chemical composition identification of BPC icon peaks.

| No. | m/z | RT/min | ppm | Adduct | score | Compound | Class |
| --- | --- | --- | --- | --- | --- | --- | --- |
| 1 | 132.1021 | 2.03 | 1.8 | [M+H-C2H2O]+ | 0.9993 | N-Acetyl-D-norleucine | Carboxylic-acids-and-derivatives |
| 2 | 132.1022 | 2.23 | 15.9 | [M+H]+ | 0.9998 | L-Norleucine | Carboxylic-acids-and-derivatives |
| 3 | 268.1043 | 2.46 | 1 | [M+H]+ | 0.9999 | Adenosine | Purine-nucleosides |
| 4 | 284.0993 | 2.62 | 1.4 | [M+H]+ | 0.9996 | Guanosine | Purine-nucleosides |
| 5 | 116.0710 | 2.78 | 3.1 | [M+H]+ | 0.8653 | Methyl 1-aminocyclopropanecarboxylate | Carboxylic-acids-and-derivatives |
| 6 | 166.0865 | 3.29 | 19 | [M+H]+ | 0.9995 | Phenylalanine | Carboxylic-acids-and-derivatives |
| 7 | 127.0393 | 3.98 | NA | NA | NA | NA | NA |
| 8 | 209.0447 | 4.54 | 2 | [M+H]+ | 0.9997 | Fraxetin | Coumarins-and-derivatives |
| 9 | 447.1290 | 5.37 | 0.6 | [M+H]+ | 0.9992 | Calycosin-7-o-beta-d-glucoside | Isoflavonoids |
| 10 | 463.0873 | 5.50 | 1 | [M+H]+ | 0.9999 | Scutellarin | Flavonoids |
| 11 | 463.0874 | 5.88 | 0.7 | [M+H]+ | 0.9981 | Kaempferol 3-glucuronoside | Flavonoids |
| 12 | 477.1033 | 6.26 | 2.6 | [M+H]+ | 0.9917 | 6-O-Methylscutellarin | Flavonoids |
| 13 | 163.0391 | 6.40 | 0.5 | [M+H-C6H6O2]+ | 0.9775 | Butein | Linear-1-3-diarylpropanoids |
| 14 | 463.0873 | 6.73 | 1.2 | [M+H]+ | 0.9978 | 6-{[2-(3,4-dihydroxyphenyl)-5-hydroxy-4-oxo-4H-chromen-7-yl]oxy}-3,4,5-  trihydroxyoxane-2-carboxylic acid | Flavonoids |
| 15 | 285.0759 | 7.92 | 1.1 | [M+H]+ | 0.9939 | Calycosin | Isoflavonoids |
| 16 | 301.0709 | 8.94 | 0.6 | [M+H-C6H10O5]+ | 0.9961 | Tectoridin | Isoflavonoids |
| 17 | 439.3574 | 9.40 | 0.5 | [M+H-H2O]+ | 0.9287 | Ursolic acid | Prenol-lipids |
| 18 | 573.3063 | 10.38 | 0.7 | [M+H]+ | 0.9662 | Ganoderic acid H | Prenol-lipids |
| 19 | 499.3058 | 10.44 | 1.2 | [M+H-H2O]+ | 0.9033 | Ganoderic acid A | Prenol-lipids |
| 20 | 117.0181 | 1.96 | 10.2 | [M-H]- | 0.9987 | Succinic acid | Carboxylic-acids-and-derivatives |
| 21 | 389.1087 | 2.42 | 0.5 | [M-H]- | 0.8106 | Secologanoside | Prenol-lipids |
| 22 | 389.1088 | 3.11 | 0.4 | [M-H-C8H8O2]- | 0.8252 | 2-(3,4-Dihydroxyphenyl)ethyl (1S,4aR,7aR)-  1-(.beta.-D-glucopyranosyloxy)-4a-hydroxy-  7-methyl-5-oxo-1,4a,5,6,7,7a-  hexahydrocyclopenta[c]pyran-4-carboxylate | Prenol-lipids |
| 23 | 197.0448 | 3.56 | 3.3 | [M-H]- | 0.9963 | (R)-3-(3,4-Dihydroxyphenyl)lactate | NA |
| 24 | 153.0183 | 3.81 | 6.6 | [M-H-H2O]- | 0.9989 | 3-Dehydroshikimic acid | Organooxygen-compounds |
| 25 | 449.1299 | 3.87 | 1.1 | [M+HCO2]- | 0.9718 | Hastatoside | Prenol-lipids |
| 26 | 771.1991 | 4.16 | 0.8 | [M-H]- | 0.8184 | 3-((6-Deoxy-4-O-.beta.-D-  glucopyranosyl-.alpha.-L-  mannopyranosyl)oxy)-2-(3,4-  dihydroxyphenyl)-5-hydroxy-4-oxo-4H-  chromen-7-yl hexopyranoside | Flavonoids |
| 27 | 137.0233 | 4.43 | 7.5 | [M-H]- | 0.9989 | 3,4-Dihydroxybenzaldehyde | Organooxygen-compounds |
| 28 | 431.1919 | 4.77 | 1 | [M+HCOO]- | 0.9071 | (3E)-4-((1S)-1-Hydroxy-2,6,6-trimethyl-4-  oxocyclohex-2-en-1-yl)but-3-en-2-yl .beta.-  D-glucopyranoside | Fatty-Acyls |
| 29 | 121.0283 | 5.23 | 8.6 | [M-H]- | 0.9993 | 4-Formylphenol | Organooxygen-compounds |
| 30 | 189.0765 | 5.36 | 3.3 | [M-H]- | 0.9112 | 3-Hydroxysuberic acid | Hydroxy-acids-and-derivatives |
| 31 | 163.0391 | 5.50 | 6.1 | [M-H]- | 0.9997 | Coumaric acid | Cinnamic-acids-and-derivatives |
| 32 | 463.0882 | 5.71 | 0.1 | [M-H]- | 0.8457 | Eriodictyol 7-O-glucuronide | Flavonoids |
| 33 | 359.0770 | 6.40 | 0.2 | [M-H]- | 0.9991 | Rosmarinic acid | Cinnamic-acids-and-derivatives |
| 34 | 187.0967 | 6.53 | 4.5 | [M-H]- | 0.9928 | Azelaic acid | Fatty-Acyls |
| 35 | 717.1458 | 6.71 | 0.3 | [M-H]- | 0.9488 | Salvianolic acid b | NA |
| 36 | 379.1759 | 6.97 | 6.7 | [M-H]- | 0.8556 | EHP(Amide) | Carboxylic-acids-and-derivatives |
| 37 | 285.0404 | 7.77 | 0.5 | [M-H]- | 0.9996 | Luteolin | Flavonoids |
| 38 | 299.0558 | 8.94 | 0.8 | [M-H]- | 0.996 | Dinatin | Flavonoids |
| 39 | 329.2332 | 9.64 | 0.4 | [M-H]- | 0.9888 | 9-Octadecenoic acid, 5,8,11-trihydroxy | Fatty-Acyls |
| 40 | 237.0553 | 11.89 | 1.8 | [M-H]- | 0.9985 | 3-Hydroxyflavone | Flavonoids |

**Table header description:** NO: serial number; m/z: parent ion mass-to-charge ratio; RT/min: retention time/min; ppm: mass accuracy; Adduct: addition of ions; Score: secondary mass spectrometry matching score; Compound EN: English name of compound; Class: classification of compound.

**Supplementary Figures**


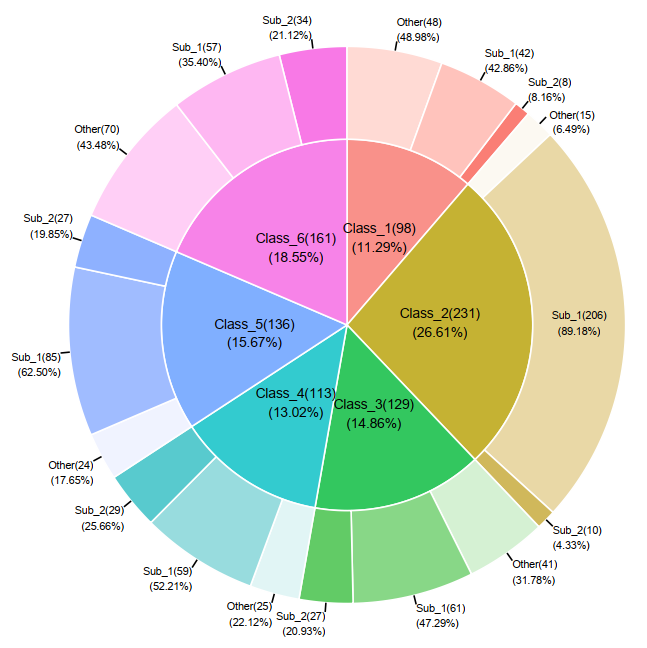


**Supplementary Figure 1.** The number of compounds in each chemical class.


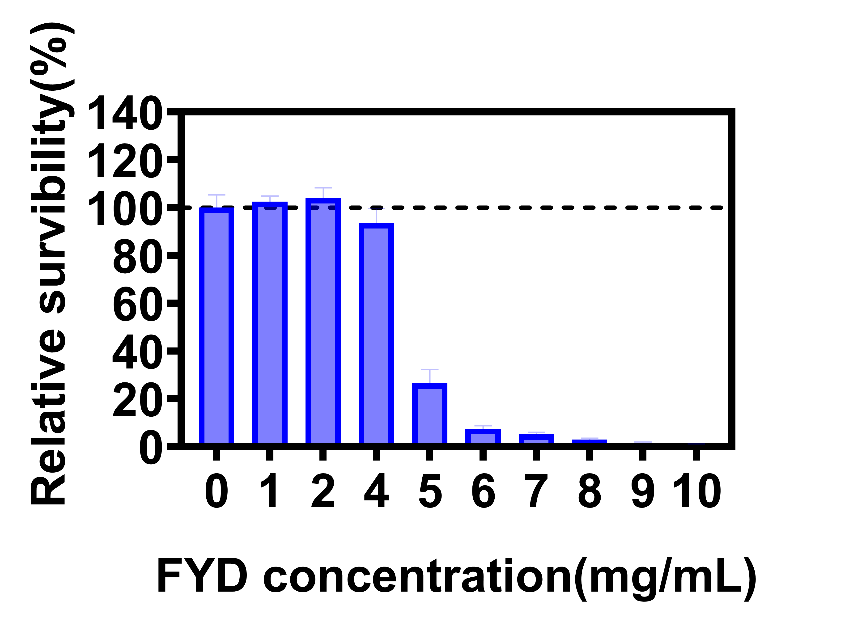


**Supplementary Figure 2.** The cytotoxicity of FYD to CNE1 cells were determined by CCK8 assay, and relative survibility was expressed as percentage of control.


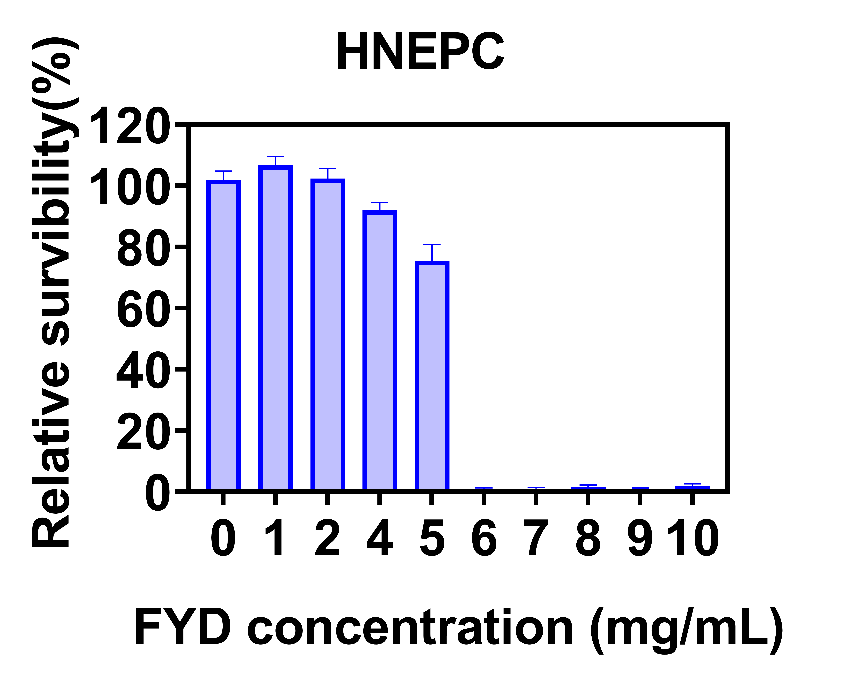


**Supplementary Figure 3.** The cytotoxicity of FYD to human nasal epithelial cells HNEPC were determined by CCK8 assay, and relative survibility was expressed as percentage of control.


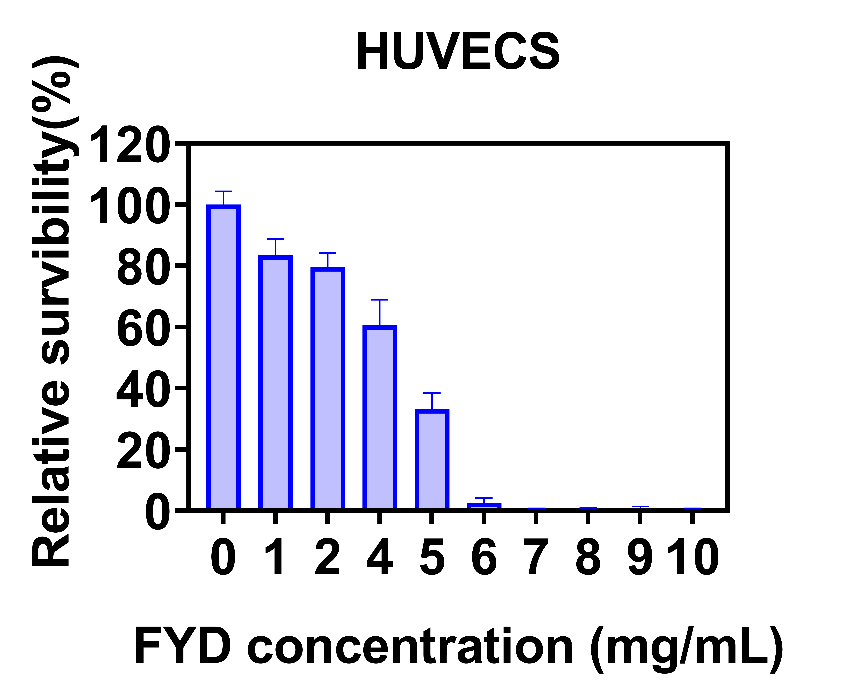


**Supplementary Figure 4.** The cytotoxicity of FYD to human umbilical vein endothelial cells (HUVECs) were determined by CCK8 assay, and relative survibility was expressed as percentage of control.


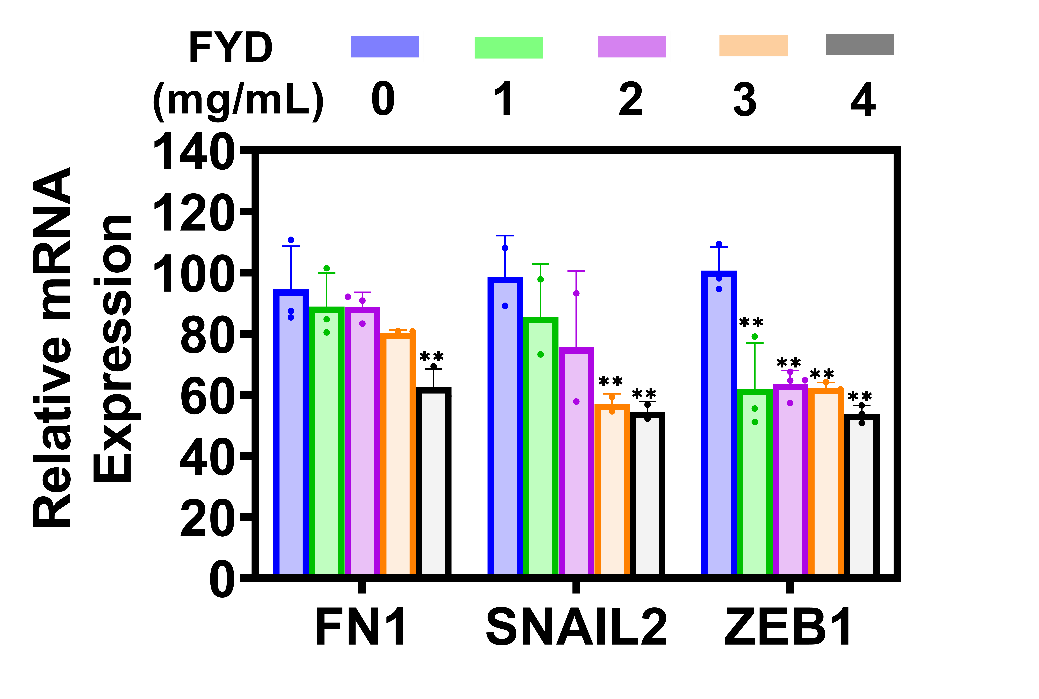


**Supplementary Figure 5.** qRT-PCR analysis showed that FYD significantly inhibited the expression of mesenchymal markers FN1, SNAIL2 and ZEB1. Data are presented as mean ± SD (n = 3); ** indicate p < 0.01 vs. Control (FYD free).


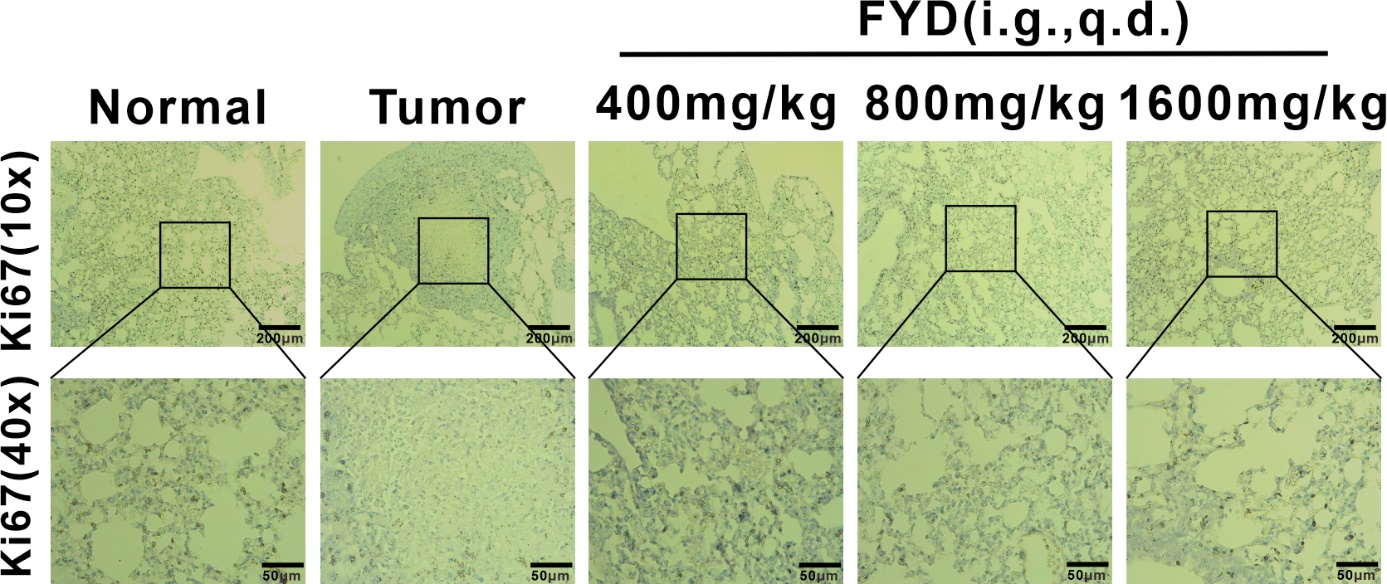


**Supplementary Figure 6.** Representative immunohistochemical staining for Ki67 in lung metastases are shown.


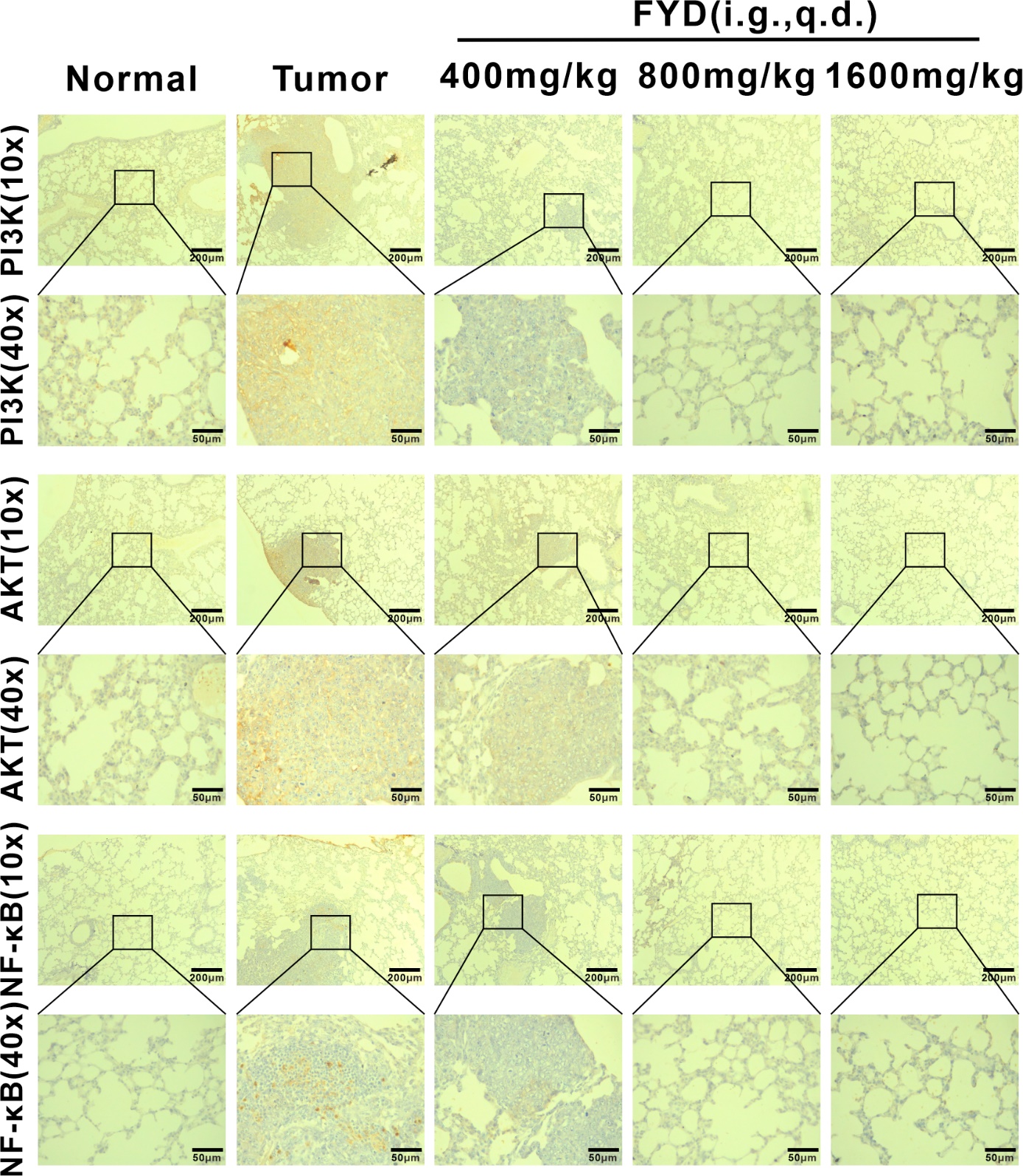


**Supplementary Figure 7.** Representative immunohistochemical staining for PI3K, AKT and NF-κB p65 in lung metastases are shown.


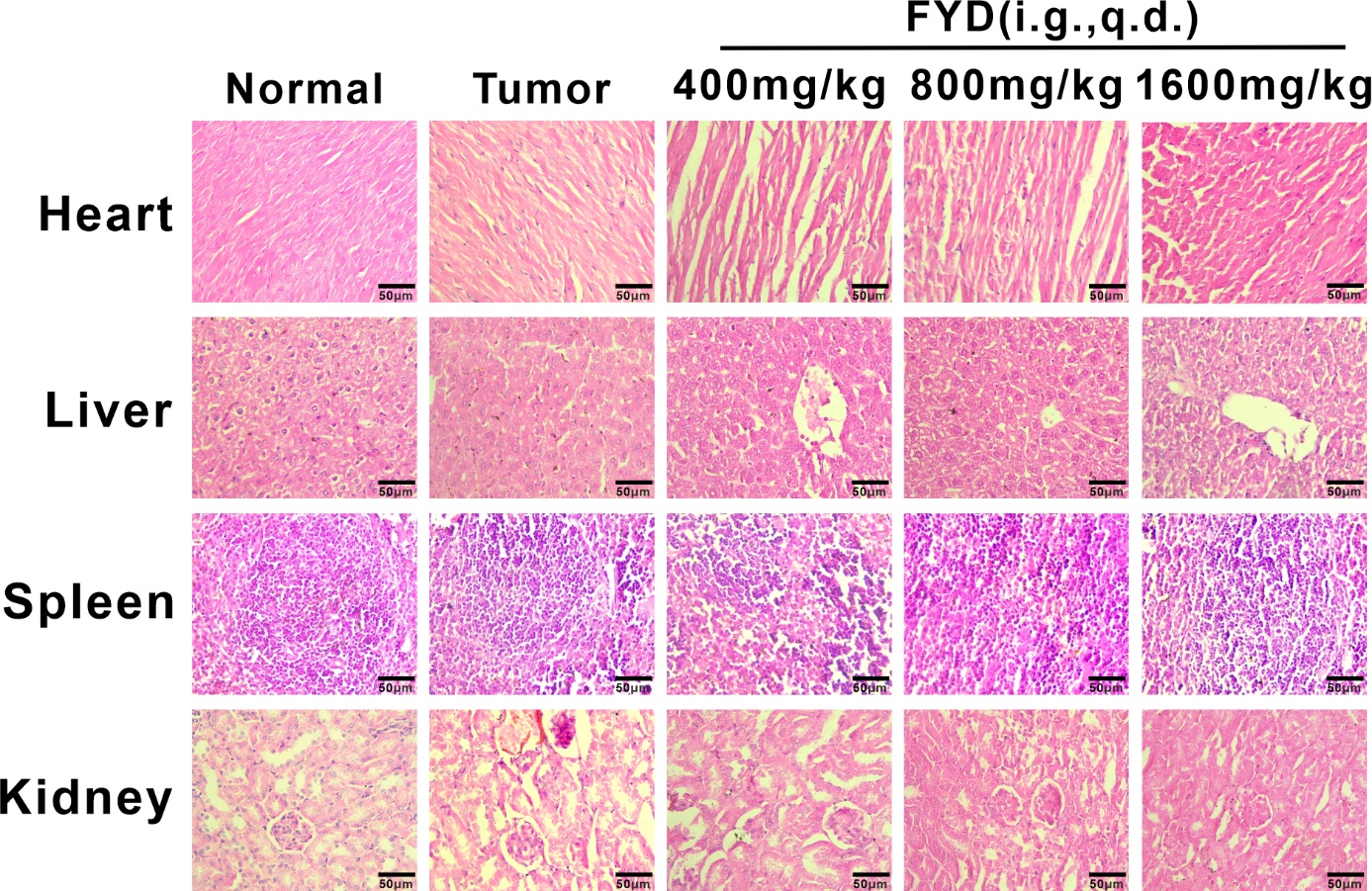


**Supplementary Figure 8.** Representative images of heart, liver, spleen and kidney sections stained with H&E from FYD-treated mice are shown.
